# Human Y chromosome sequences from Q Haplogroup reveal a South American settlement pre-18,000 years ago and a profound genomic impact during the Younger Dryas

**DOI:** 10.1371/journal.pone.0271971

**Published:** 2022-08-17

**Authors:** Paula B. Paz Sepúlveda, Andrea Constanza Mayordomo, Camila Sala, Ezequiel Jorge Sosa, Jonathan Javier Zaiat, Mariela Cuello, Marisol Schwab, Daniela Rodríguez Golpe, Eliana Aquilano, María Rita Santos, José Edgardo Dipierri, Emma L. Alfaro Gómez, Claudio M. Bravi, Marina Muzzio, Graciela Bailliet

**Affiliations:** 1 Instituto Multidisciplinario de Biología Celular, Universidad Nacional de La Plata, Consejo Nacional de Investigaciones Científicas y Técnicas, Comisión de Investigaciones Científicas, La Plata, Buenos Aires, Argentina; 2 Programa de Cáncer Hereditario, Hospital Italiano de Buenos Aires, Ciudad Autónoma de Buenos Aires, Buenos Aires, Argentina; 3 Instituto de Química Biológica de la Facultad de Ciencias Exactas y Naturales, Facultad de Ciencias Exactas y Naturales, Universidad de Buenos Aires, Consejo Nacional de Investigaciones Científicas y Técnicas, Ciudad Autónoma de Buenos Aires, Buenos Aires, Argentina; 4 Instituto de Biología de la Altura, Facultad de Humanidades y Ciencias Sociales, Universidad Nacional de Jujuy, San Salvador de Jujuy, Jujuy, Argentina; 5 Instituto de Ecorregiones Andinas, Universidad Nacional de Jujuy, San Salvador de Jujuy, Jujuy, Argentina; 6 Facultad de Ciencias Naturales y Museo, Universidad Nacional de La Plata, La Plata, Buenos Aires, Argentina; Universitat Pompeu Fabra, SPAIN

## Abstract

The settlement of the Americas has been the focus of incessant debate for more than 100 years, and open questions regarding the timing and spatial patterns of colonization still remain today. Phylogenetic studies with complete human Y chromosome sequences are used as a highly informative tool to investigate the history of human populations in a given time frame. To study the phylogenetic relationships of Native American lineages and infer the settlement history of the Americas, we analyzed Y chromosome Q Haplogroup, which is a Pan-American haplogroup and represents practically all Native American lineages in Mesoamerica and South America. We built a phylogenetic tree for Q Haplogroup based on 102 whole Y chromosome sequences, of which 13 new Argentine sequences were provided by our group. Moreover, 1,072 new single nucleotide polymorphisms (SNPs) that contribute to its resolution and diversity were identified. Q-M848 is known to be the most frequent autochthonous sub-haplogroup of the Americas. The present is the first genomic study of Q Haplogroup in which current knowledge on Q-M848 sub-lineages is contrasted with the historical, archaeological and linguistic data available. The divergence times, spatial structure and the SNPs found here as novel for Q-Z780, a less frequent sub-haplogroup autochthonous of the Americas, provide genetic support for a South American settlement before 18,000 years ago. We analyzed how environmental events that occurred during the Younger Dryas period may have affected Native American lineages, and found that this event may have caused a substantial loss of lineages. This could explain the current low frequency of Q-Z780 (also perhaps of Q-F4674, a third possible sub-haplogroup autochthonous of the Americas). These environmental events could have acted as a driving force for expansion and diversification of the Q-M848 sub-lineages, which show a spatial structure that developed during the Younger Dryas period.

## Introduction

The human settlement of the American continent has been subject of extensive debate and controversy in the academic community for more than 100 years [1, 2]; central questions related to the time of arrival as well as the spatial distribution patterns are still open and under discussion. The scenario mostly accepted by both archaeological and genomic studies is the one that proposes a settlement of the American continent with an intermediate chronology between ~18,500 to 13,000 calibrated years before present (cal BP) and a human entry in South America shortly after [3–8]. Moreover, other controversial hypotheses suggest a longer chronology with a South American settlement before 18,000 cal BP [9–15].

The human Y chromosome is used as a highly informative tool to investigate the history of human populations, since it has the longest stretch of non-recombinant DNA in the entire human genome, and is completely transmitted from fathers to sons, containing a record of the history of the paternal lineage [16]. Advances in the complete Y chromosome sequencing technique provided by next-generation sequencing (NGS) platforms have made it possible to create robust *de novo* phylogenetic trees based on a large set of mutational data, where the branch lengths are proportional to the number of SNPs and, therefore, to time [17]. These studies are proving valuable in providing information and new insights into human male history. Q Haplogroup in the Y chromosome is the only Pan-American haplogroup and represents virtually all Native American lineages in Mesoamerica and South America [6]. The autochthonous Q-M3 sub-haplogroup of Amerindians has been previously described at high frequency [18] and with a star-shaped phylogenetic topology that has been interpreted as the initial colonization of South America with a rapid expansion ~15 thousand years ago (kya) [7]. Furthermore, it has been observed that Q-M848 sub-lineages (within Q-M3) present a spatial structure in South America that arose as early as ~12.3 kya [5]. Q-Z780 is another Native American autochthonous sub-haplogroup which occurs at low frequency [19] and is still little studied from genomic data due to its low availability in sequence databases. A recent report using high coverage complete sequences has dated this lineage ~17 kya, which was explained as a more complex settlement scenario in the Americas where the deep branches could reflect a separate out-of-Beringia dispersal after the melting of the glaciers at the end of the Pleistocene [5].

In order to have a more integrated view about the settlement of the Americas it is necessary to analyze the environmental changes that influenced the human populations of the time. The Younger Dryas (YD) was a major large-scale rapid climate change detected in the Northern Hemisphere about 12,900–11,600 cal BP [20, 21]. The Younger Dryas impact hypothesis at 12,800 cal BP has been associated with the abrupt YD climatic changes, large-scale megafauna’s extinction and decline and/or reorganization of human populations [22–24].

To provide new insights into the history of American settlement, we present here a complete phylogenetic reassessment of the Y chromosome Q Haplogroup. For the first time in a genomic study of Q Haplogroup, current knowledge on Q-M848 sub-lineages is contrasted with the historical, archaeological, and linguistic data available. We propose a hypothesis for the American settlement based on the divergence times of the Native American lineages and recent archaeological research.

## Results and discussion

We generated new whole Y chromosome sequences from 13 Argentine samples belonging to Q Haplogroup (see Table A in S1 Text), non-randomly chosen to cover the extent of known Y short tandem repeats (STR) diversity [25]. We compared these to 89 samples published worldwide, belonging to Q Haplogroup, including 76 American and 13 Eurasian Y chromosome sequences, resulting in a combined dataset of 102 Y chromosome sequences (for more information about the dataset, see S1 Table).

**Fig 1:**
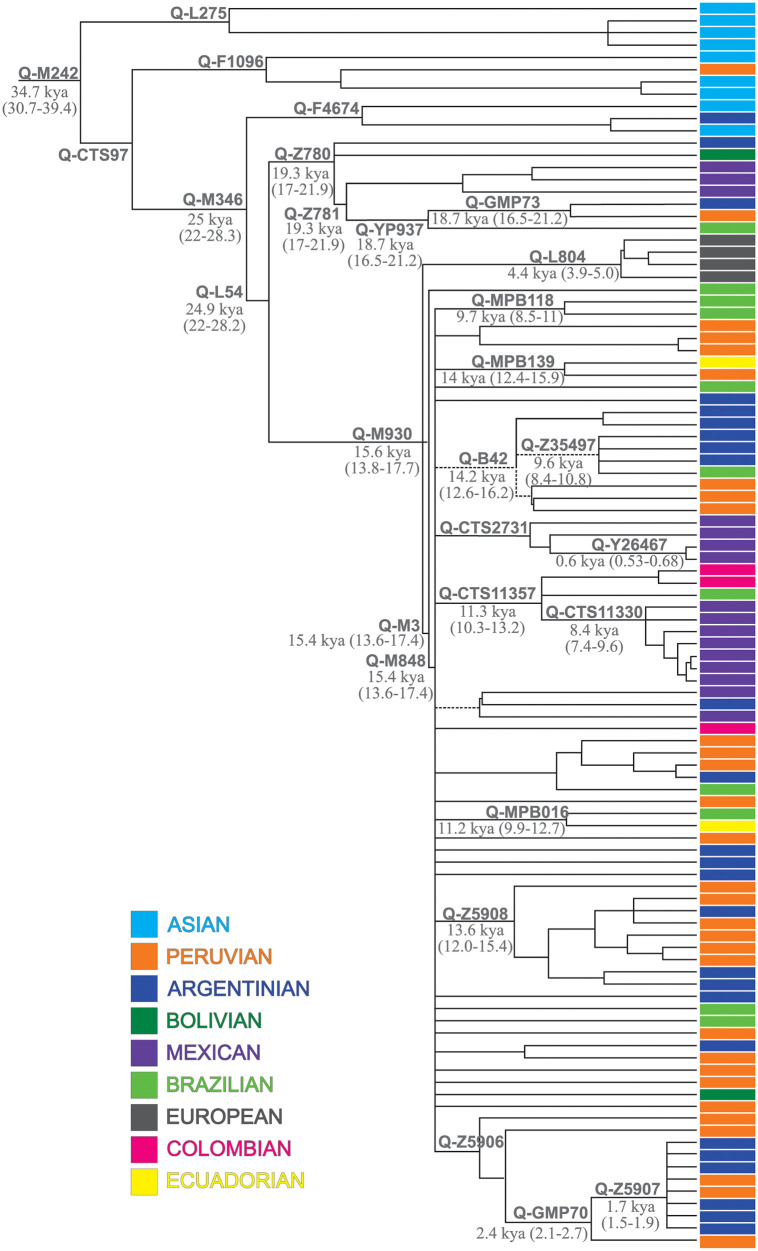
Q-M242 haplogroup phylogeny reconstructed with 102 Y chromosome sequences. The colors in the tree box represent each individual and are in accordance with the macro-area or the country according to the box on the left. Divergence times estimated in this study are represented in italics, in kya, and with a 95% confidence interval between parentheses (for more details see Methods). Dashed lines are used to represent branches that require further study for a better definition. For more information on data set, see Table A in S1 Text and S1 Table, and for more detailed phylogenetic representation, see S5 Fig.

We analyzed SNPs from 10.45 Mb regions within which reliable genotype calls can be made in the non-recombinant region of the Y chromosome (NRY) [26], covered in all 102 modern sequences of this study, resulting in 8,839 high-confidence SNPs (see Methods and S1 Text). We updated the phylogeny for Q Haplogroup, which includes new divergence times estimated in this work (see Fig 1 and S5 Table). Taking all the sequences from this study into account, 19 previously known Q sub-haplogroups were defined; of these, 1 belongs to L275, 1 to F1096, 1 to Q-F4674, 1 to Q-Z780, 1 to L804, and 14 to Q-M848. Moreover, we present here 1,072 novel informative SNPs absent in ISOGG (S2 Table) out of which 74 SNPs were validated and named as Q-GMP1 to Q-GMP74 (see S3 and S4 Tables). GMP51, GMP73 and GMP74 were able to define two new sub-lineages (See S1, S2 Tables and S5 Fig). These new data provide further support and vastly increase the resolution of Q Haplogroup. Seven of our 13 Argentine sequences presented in this work allowed us extending the sub-haplogroups resolution, of which 4 belong to Q-M848, 2 to Q-Z780, and 1 to Q-F4674 (see S5 Fig and S1 Table). Moreover, 6 of them combined with 13 other sequences from databases are part of phylogenetic branches that are currently polytomic.

In the following sections, we will discuss the novel results, including the necessary background for a better understanding of our conclusions. The lineages studied in depth in other reports such as: Q-L275 distributed in Eurasia [27]; Q-F1096 in Eurasia, in Athabaskan natives from North America, in Greenland, in ancient Aleutian Islanders, and in ancient Northern Alaskan Athabascans [16, 28–33]; and Q-L804 in Northern Europeans [34, 35], are not analyzed here.

### Sub-lineage Q-M346

Q-M346 was dated 25 kya (22–28.3) in the present work, within the range previously reported in literature [5]. It has been described in Eastern Europe, Middle East, Central Asia, Eastern Asia, Southern Siberia, and in the Americas [18, 32, 36–38]. The most prevalent Native American lineages, Q-Z780 and Q-M3, are derived from Q-M346 [18]. It is known that one of its bifurcations occurs for Q-B28 and Q-L54 [7].

Q-F4674 is a sub-lineage of Q-B28, the latter currently with no clear location in ISOGG [39], though it is the marker most widely described in the literature for this lineage. Q-B28 occurs in Europe and in South and East Asia [6, 39]. Q-F4674 was previously dated 18.3 kya (16.7–19.9) [40]. One of the new Argentine samples sequenced in this study, from San Juan (RUTBE), is presented as a sub-lineage of Q-F4674 along with 2 other Sri Lankan samples from the databases (S5 Fig and S1 Table). In turn, San Juan’s sample shares Q-Z36057 with 1 of Sri Lanka’s individuals; the age of this lineage has not been yet estimated. The occurrence of Q-F4674 in the Americas has recently been found and published by our group [38]. In the aforementioned study, 2 samples from San Juan, including RUTBE are presented as Q-M346* (derived for Q-M346 and ancestral for Q-L54), very differentiated from each other, and not parentally related. In the present study, we verified that both San Juan’s samples have Q-F4674 and Q-Z36057. These new results lead us to reinforce what was previously proposed by Jurado Medina et al. [38]: Q-M346*, more precisely Q-F4674, would be a third autochthonous sub-haplogroup of the Americas, along with Q-M848 and Q-Z780. Q-F4674 could be part of the gene pool of ancient founding Native American paternal lineages but may not have had as much survival success as Q-M848 and Q-Z780 sub-lineages [38, 41] (possible reasons for this low frequency are discussed in the Hypothesis on the American Settlement section of this work). We believe that a higher number of individuals derived for Q-M346* could be found in the American continent, but since studies on Native American lineages that have included Q-M346 have not analyzed Q-L54 [42], Q-M346* sub-lineages become difficult to be registered.

### Sub-lineage Q-Z780

Q-Z780 haplogroup is recognized as a Y chromosome founding lineage in the Americas at low frequency [43]. It is widely distributed, with representatives from Mexico, Peru, Bolivia, Brazil, and Argentina in this study, but its presence has been also reported for Central America, Colombia and Paraguay [6, 19, 36, 38]. Given its low frequency and scarce sequence availability in databases, little is known about its sub-lineages. According to the best known markers, it can be classified into Q-Z781 and Q-FGC47532 [39], the latter characterizing the ancient Anzick-1 Y chromosome (with a 14C calibrated age of 12,600 cal BP) [44].

Four new SNPs parallel to Q-Z780 not described in ISOGG were found; one of them was validated and named Q-GMP10 (see S2 and S4 Tables). Two samples incorporated in this study to Q-Z780 (Z8ZMY and S8BAL) allowed adjust its temporal depth, showing values of 19.3 kya (17–21.9), older than those reported in the literature of 17 kya (15, 0–19.3) [5]. Two samples within Q-Z780 are polytomic, 1 of them is a sample from our collection (Z8ZMY) which has 62 private SNPs absent in ISOGG that provide new information to this lineage, and 2 of them were validated as Q-GMP13 and Q-GMP14 (see S2 and S4 Tables).

### Sub-lineage Q-Z781

Q-Z781 is the most represented sub-lineage of Q-Z780. Given the similar number of Q-Z780 and Q-Z781 sequences, the dates found for both are equal, though older than those found by other authors with values of 16 kya (14.1–18.1) for Q-Z781 [5]. However, older dates as 22.9 kya (18.3–27.5) have been found from STRs for a great number of samples for this sub-lineage [6]. Q-Z781 branches into Q-Y2816 and Q-YP937. Q-Y2816 is distributed mainly in individuals of Mexican origin [7, 45], and also in an individual from the United States without a defined origin [34]. We found 3 individuals of Mexican origin in this sub-lineage, 2 of which also share Q-Z782.

Q-YP937 is characteristic of South America with a wide distribution from Peru and Argentina to Brazil. We found 3 new SNPs parallel to Q-YP937 (S2 Table). The dating for this sub-lineage is 18.7 kya (16.5–21.2), which is older than that of 12.5 kya (11–14) reported in literature [6]. We found a new sub-lineage, not described in ISOGG, supported by 2 SNPs named Q-GMP73 and Q-GMP74 (see S6 Fig, S2 and S4 Tables) with a dating of 18.2 kya (16.1–20.6). The phylogenetic association found for this new sub-lineage evidences a link between Andean individuals and Central-West Argentina with dates for which there are no archaeological records showing such temporal depth for human groups from this region. Further analysis of these findings is provided in the Hypothesis of the American Settlement section.

### Sub-lineage Q-M3

Q-M3 haplogroup has been previously described as a founder lineage of the Y chromosome in the Americas [46–48] and is the most frequent sub-lineage among present-day Native Americans [18, 49–51]. Although its presence has also been described for some populations from Siberia, it is not known whether these are remains of the founding lineage or evidence of regressive migrations from Beringia to East Asia [37]. The dating found for this marker in the present work is 15.4 kya (13.6–17.4), within the range previously reported in the literature [5].

In recent decades, the internal resolution of Q-M3 has expanded and this lineage is now known to be subdivided into two branches, Q-M848 and Q-Y4308 [5, 6]. Although Q-Y4308 is still underrepresented, it is widely distributed. Its presence has been reported for individuals from the United States in association with those who speak the Algonquian language [52], Eskimo peoples from the extreme Northeast of Asia [52], Mexican individuals [53], and a Tupi-Guarani individual from Southern Brazil reported in this work (S5 Fig), this latter in agreement with previously reported data [5].

Q-M848 is the most represented sub-lineage of the Q Haplogroup in the Americas, and more frequent in South America than in North America. It has been previously described with a star-shaped topology where many short branches are connected in the same internal node (S5 Fig) [5–7]. Given the high Q-M848 and poor Q-Y4308 representativeness of the samples studied in this work, the datings for Q-M848 and Q-M3 show the same values, within ranges estimated in the literature [5]. The fossil remains of Kennewick man [54], found on the banks of the Columbia River in the United States, belong to Q-M848 haplogroup and have been dated 8.3–9.2 cal BP [5, 55].

### Sub-lineage Q-MPB118

The Q-MPB118 sub-lineage was found here defining the same Aranã samples from Southeast Brazil and Xavante from West Brazil (S6 Fig and S1 Table), in agreement with previous reports [5]. For the moment, this lineage is restricted to Brazilian individuals [56]. We found 6 new SNPs not validated, provided in this work as new information to this lineage (S2 Table). The dating found here for this node is 9.7 kya (8.5–11) (S5 Table), similar to previously reported estimates [5]. Since the aim of the present study is to reconstruct the history of the lineages belonging to Q-M848, in section 6 of S1 Text, we present further information for each of these sub-lineages regarding the history of their ethnic groups, linguistic family, and the region they inhabit or inhabited.

Q-MPB118 supports a lineage ancestry shared between native Xavante and Aranã groups, both of the Macro-Jê linguistic trunk. Since its differentiation (~9.7 kya), this lineage is present among human groups from Central-West and Southeast Brazil, although further study on its distribution is still necessary. For a schematic representation of the geographic distribution of Q-MPB118, see S4B Fig.

### Sub-lineage Q-SK281

Q-SK281 is currently presented as a restricted lineage for Peruvian individuals (S6 Fig), dated 12.6 kya (12.1–13.1) [57]. This study provides 19 new SNPs for its sub-lineage defined by Q-Z35727 (S2 Table). For a schematic representation of the geographic distribution of Q-SK281, see S2C Fig.

### Sub-lineage Q-MPB139

Uro samples from Peru and Pasto from Ecuador were found here supported by Q-MPB139 in agreement with previous reports [5]. The dating found in the present study was 14 kya (12.4–15.9), similar to the one previously reported [5].

Q-MPB139 shows a shared lineage ancestry between the Uros of Peruvian Altiplano and the Pasto of the Ecuadorian Altiplano, evidencing a great temporal depth and vast movements of human groups between the Central and North Andean Areas, near 14 kya (12.4–15.9). For a schematic representation of the geographic distribution of Q-MPB139, see S1A Fig. In this lineage the Uro individual is separated from other characteristic lineages of Peruvians and individuals of the Central Andes (such as: Q-SK281, Q-Z6658, Q-Y788, Q-Z5908, Q-Z35841, and Q-Z5906), providing genetic support to anthropological and linguistic hypotheses that consider the Uro ethnic group different from neighboring ethnic groups (such as Aymara and Quechua), with its own language, traditions, beliefs, and ways of hunting, fishing, gathering and farming [58, 59]. Previous studies carried out with Y chromosome microsatellites found that the Uros have exclusive lineages different from Aymara, Quechua, and Arawak haplotypes [42], whom they have been associated with in other studies [60]. According to some researchers, the Uros were the first settlers of the Andean Altiplano; however, their origin is unknown and is currently subject of academic debate [58, 61–64].

### Sub-lineage Q-B46

Q-B46 has been previously described as characteristic of Colla individuals from Salta [8, 52], which we have corroborated in this study even extending its distribution to Northwestern Argentina due to its presence in a sample from this region (S5 Fig and S1 Table). In the present work we contribute 2 new SNPs absent in ISOGG equivalent to Q-B46, validated as Q-GMP21 and Q-GMP22. Moreover, we found 100 new SNPs not reported in ISOGG private for the Catamarca’s sample of our collection and without data for the Colla’s sample from the databases. Three of these SNPs were validated as GMP23 to GMP25 (see S2 and S4 Tables).

Although this lineage still has very few sequences and its dating has not been established, its phylogenetic relationship is expected to be found since the autochthonous peoples of the Northwestern region of Argentina have remained permanently related to each other since ancient times, through exchange, trade, migration, and the promotion of their artistic and craftwork materials [65].

### Sub-lineage Q-Z35505 / Q-Z35497 / Q-B43

So far, the Q-B43 sub-lineage has been described by other authors in Wichi individuals from Salta, Argentina [8], and in individuals from Paraguay and Brazil [6].The present study includes an individual from the Paresí community, from Mato Grosso, Brazil, obtained from the databases [5], as well as another individual from Salta, from our collection.

The phylogenetic relationship found in this work for 3 individuals from East Salta, Argentina, was supported by Q-Z35505, parallel to Q-B43 [39]. Moreover, one of these samples from the East of Salta, from our collection, shares 26 SNPs with the individual from the Paresí community, out of which Q-Z35497 is also parallel to the 2 markers mentioned above [39]. We contribute 5 new SNPs to this sub-lineage (S2 Table), 4 of them validated as Q-GMP26 to Q-GMP29 (S4 Table). We also found 50 new private SNPs for our Salta sample of this lineage (S2 Table), 4 of them validated as Q-GMP30 to Q-GMP33 (S4 Table). The dotted line in S5 Fig for this sub-lineage means that further studies are required for its better definition; here we have found some difficulties due to the large amount of missing data present in samples for which complete sequences are not available and are in VCF format (see Section 3 in S1 Text and S1 Table).

The dating of this lineage had been previously estimated as 1.5 kya (0.9–2.1) [6], calculated only between 2 samples (GS000016946-ASM and GS000016945-ASM) for which the complete sequence is not available and comes from VCF files (see section 3 in S1 Text and S1 Table). The dating found in this work, calculated only between two samples for which the complete sequence is available and present greater sequencing coverage (N87FK8 and GRC14349596_S) (see section 2 in S1 Text and S1 Table), is 9.6 kya (8.4–10.8). We believe that our results could be reflecting a temporal estimate more in line with the greater geographic distribution found here for this lineage. On the other hand, the estimate of ~1.5 kya [6] could reflect some internal sub-lineage with regional differentiation in Argentina’s Northwest and also characteristic of the Wichi community; this could be better defined in the future with the incorporation of more samples to this lineage.

In this study we present genetic evidence that associates within the same sub-lineage (Q-Z35505/Q-Z35497) Mataguayan-speaking individuals from Gran Chaco and Arawak-speaking individuals from the Mato Grosso region, bordering the Gran Chaco (for more information see section 6 in S1 Text). In this regard, it has been previously argued that Mataguayan-speaking population may have moved to the Southeast due to pressure from Amazonian groups, speaking Arawak languages [66]. In fact, some sort of exchange must have taken place between Mataguayos and local Arawak farmers before their settling in the area, since some archaeological sites in the Gran Chaco reveal similar but more rudimentary decorated pottery [67]. We present genetic support for these hypotheses, adding a temporal depth for Q-Z35505/Q-Z35497 of ~9.6 kya. We cannot determine whether Mataguayan-speaking and Arawak-speaking communities have a common origin or if both groups have different origins and then linked and admixed leaving shared genetic traits. The dates found suggest that Gran Chaco could have been inhabited earlier than estimated [68].

### Sub-lineage Q-Z6658 / Q-Z5915

This lineage is currently restricted to individuals from Peru and has been previously described [5, 6, 69]. A dating of 12.0 kya (9.5–14.7) was found in literature [69].

### Sub-lineage Q-B42

The marker Q-B42 has been previously described as ancestral to Q-B43 (parallel to Q-Z35505) and Q-B46 [8]. Q-B42 is known to be a recurrent mutation that is used to describe another sub-lineage belonging to the European R haplogroup (R1b1a1b1a1a2c1a3a2a1d3). Given this characteristic, the ISOGG platform does not include this marker within Q Haplogroup, but it is still used in current works on the phylogenetic reconstruction of Q Haplogroup [6]. In the present study, Q-B42 is present among individuals belonging to the sub-lineages Q-B46, Q-Z35505, and Q-Z6658 (discussed above) but it is absent in some individuals within the last two sub-lineages (see S1 Table). S5 Fig proposes the position of Q-B42 based on these results, which is represented with a dotted line suggesting that findings should be further studied. The contribution of two new high coverage complete sequences provided in this work belonging to Q-B42 allows their dating to be adjusted to values of 14.2 kya (12.6–16.2), older than those of 10.1 kya (8.4–11.8) found in the literature [6].

Q-B42 occurs among individuals from Peru, Northwest Argentina, and Central Chaco. For the Huaca Prieta archaeological site located near the Pacific coast in Northern Peru, radiocarbon dating indicates intermittent human presence between ~15,000 and 8,000 cal BP [70]. In Northwestern Argentina, several sites date from ~12.0 kya and possibly as early as ~12.8 kya [71].

As proof of the influence of the first civilizations of the Andean highlands on Northwestern Argentina, such as the pre-Inca culture of Tiwanaku located near Titicaca Lake within the current territory of Bolivia, cultural legacy has been found in Peru, Chile, and elsewhere Northwestern Argentina [72]. The Collasuyu was part of the Inca Empire (Tawantinsuyu) and expanded to the Argentine Northwest [73]. Q-B42 could have differentiated in the central Andean region and could be one of the oldest Q-M848 sub-lineages with 14.2 kya (12.6–16.2), being part of the gene pool of cultures that settled in this region. This sub-lineage shows links and gene flow among Andean, Chaco, and Amazonian groups, in accordance with archaeological studies that have found cultural evidence showing that Chaco human groups have received peripheral influence, both Andean and Amazonian [74]. The phylogenetic relationships found in the present study for Q-B42 and Q-Z35497 provides genetic support to these findings. The characteristics of the Chaco territory, with fluctuating seasonality in relation to the flood levels of the land, may have not been an obstacle for a constant interrelation among the human groups of these regions. For a schematic representation of the geographic distribution of Q-B42 and its sub-lineages, see S1C Fig.

### Sub-lineage Q-CTS2731

The Q-CTS2731 lineage is currently restricted to native populations of the United States and Mexico [52, 75]. We have found this lineage in 4 Mexican samples from the databases (S1 Table), in agreement with previous reports [5, 6]. The estimates found in the literature for this lineage are 12.4 kya (10.6–14.3) [75]. We contribute 2 new SNPs parallel to Q-CTS2731; 2 new SNPs parallel to the Q-CTS8571 sub-lineage; and 43 new SNPs parallel to the Q-Y26467 sub-lineage, absent in ISOGG (S2 Table).

Q-Y26467 has been described as characteristic of the Zapotec male population of Mexico [6, 52], which has also been observed in the present study (S1 Table). The dating found in this work for this node is 0.6 kya (0.53–0.68), within the range previously reported [6, 76].

The timing found for the first human occupation in Mexico is currently under discussion. Archaeological studies consider that Mexico shows consistent evidence of human occupation from at least 40–30 kya [15, 77–81], but dating for that period is controversial. Yet, there is not so much discussion about the common scattered sites in Mexico for the 10.5–13 kya period [80, 81].

It is known that the writing style used in the Central Valleys of Oaxaca, belonging to the Zapotec scribal tradition, constitutes the earliest evidence of writing in the American continent. The first tangible manifestations of the graphic system can be dated to approximately 600 years before the Common Era [82]. These archaeological dates are consistent with those found for the Zapotec sub-lineage Q-Y26467. For a schematic representation of the geographic distribution of Q-CTS2731 and its sub-lineages, see S2A Fig.

### Sub-lineage Q-CTS11357 / Q-M925

This lineage is known to have a wide distribution beginning at Southwest United States and extending through Mexico, Central America, and South America [5, 6, 52, 83]. In the present study, Q-CTS11357 is widely represented by 7 individuals from Mexico, 2 from Colombia, and one from Brazil, in accordance with previously reported data [5, 6]. The dating found in this work for this lineage is 11.3 kya (10.3–13.2), close to that found by other authors [5, 6, 83]. We contributed 4 new SNPs to Q-CTS11357 sub-lineages, absent in ISOGG (S2 Table).

Q-CTS11357 is classified into the following sub-lineages:

Q-Z5917 found in individuals from Colombia in the present study and in individuals from Panama and Nicaragua from the databases [6, 84].

Q-SK1974 (equivalent to Q-Y26547) [39] is currently restricted to individuals from Brazil; in fact, we found this sub-lineage in a Brazilian individual from the database, belonging to the Karitiana ethnic group [5, 6, 85].

Q-BZ4012 is at the moment restricted to individuals from the United States [83], is absent in ISOGG, and no data are reported for this sub-lineage in this study.

Q-CTS11330 has been described in this and other research works as characteristic of Mexican individuals [5, 6] though has been also found in one individual from San Salvador de Jujuy [83]. The dating found in this study for Q-CTS11330 is 8.4 kya (7.4–9.6), close to the literature estimates [6].

The Q-CTS11357 lineage is represented by individuals of the Pima and Nahua, speakers of the Uto-Aztecan language, and Karitiana ethnic groups, speakers of the only remnant of the Arikém linguistic family, being a sub-family of the Tupí linguistic trunk (see S1 Table and section 6 in S1 Text). For a schematic representation of the geographic distribution of Q-CTS11357 and its sub-lineages, see S3B Fig. The Q-CTS11357 lineage shows that approximately 11.3 kya (10.3–13.2) there was a population focus in Mexico that extended to Southwest United States, Central America, reaching Colombia and the Brazilian Amazon. At present, this lineage finds greater representation and differentiation in Mexico, with Uto-Aztecan speaking representatives (See S1 Table). This evidence could provide genetic support to previous hypotheses suggesting that the Proto-Uto-Aztecan speaking community could have formed in Central Mexico, being one of the drivers of the primary domestication of maize [86, 87]. Its expansion towards North America and the Amazon could have been driven by demographic pressure resulting from a growing commitment to the cultivation of this gramineous. The phylogenetic links found for this lineage are also in agreement with studies on the genetic diversity of corn using contemporary and archaeological maize samples [88], showing that corn used by Brazilian indigenous populations, including those from the Amazon, is genetically closer to corn samples from Mexico, as compared to other regions such as the Andes. Q-CTS11357 evidences a shared lineage ancestry between Uto-Aztecan- and Arikém-speaking human groups; given its temporal depth it is likely that this lineage has formed part of the gene pool of both the proto-Uto-Aztecan and proto-Tupí speakers. It is not possible to define whether both groups have a common origin or, having different origins, left shared genetic traits due to their geographic expansion.

### Sub-lineage Q-Y27993/Q-Y27992

Currently, this lineage has been reported for individuals from Mexico and Argentina [6, 89]. The ISOGG platform defines Q-Y27993 and Q-Y27992 as parallel; we have found Q-Y27993 in a Mexican sample from the databases and an Argentine individual belonging to the Chané ethnic group from Salta (S1 Table). Q-Y27992 occurs in a Mixtec individual from Oaxaca, but we have not found any marker shared by the three samples; therefore, in Fig 1 and S5 Fig it is represented with dotted lines since further study is needed to determine this link.

The time estimate found for this lineage is 16.1 kya (14.2–18.2) (see S5 Table), older than that found in the literature [6, 89]. We consider that the current dating calculations for this lineage are subject to biases due to low sample size and lineage resolution. If the three samples were not a monophyletic group and therefore Q-Y27992 and Q-Y27993 would not belong to the same sub-lineage, then the dating found in this study would be an error. On the contrary, if they really are a monophyletic group, our results could indicate that it is one of the oldest lineages of Q-M848, and this would lead to question its dating. However, given that the status of Q-Y27993/Q-Y27992 still requires further studies and higher resolution, in S5 Fig we consider the dating calculated in the literature as 12.6 kya (12.1–13.1) [89]. For a schematic representation of the geographic distribution of Q-Y27993/Q-Y27992, see S2B Fig.

Q-Y27993 occurs among individuals from Mexico and Chané from Northern Argentina. The language spoken by the Chané belongs to the Arawak linguistic group, one of the largest and most dispersed linguistic families in the Americas [90]. Q-Y27993 provide genetic support to the links found between Arawak-speaking individuals and Mexican communities, though further studies would be necessary to determine the ethnic relationship found between these regions.

### Sub-lineage Q-Z19357

This lineage has been reported before in Peru [91] and in Argentine individuals of the Colla ethnic group from Salta province [6]. In this study we corroborate this previous distribution and add a Brazilian individual from a database belonging to Maxakalí ethnic group from Minas Gerais, as a new contribution to this sub-lineage (S1 Table and S5 Fig). For a schematic representation of the geographic distribution of Q-Z19357 and its sub-lineages, see S3C Fig.

Q-Z19357 provides evidence of a shared lineage ancestry among individuals from Andean Peru, Northwestern Argentina, and the Brazilian Maxakalí ethnic group with a temporal depth of 8.1 kya (9.5–6.7), as reported in the literature [6]. We cannot determine whether these groups have a common origin or have different origins and were later linked and admixed leaving shared genetic traits. The greatest current diversity for this lineage occurs among Andean individuals, so it is likely that this sub-lineage has differentiated among Andean human groups, perhaps within the current territory of Peru, a region known for being the cradle of great South American civilizations, expanding its ties with the Macro-Jê-speaking communities of Brazil, native language of Maxakalí groups.

These people have probably also established relationships with Chaco human groups because this area relates the Andean region with the Brazilian Cerrado Ecoregion, which was extensively inhabited by Macro-Jê speakers in times previous to European colonization. In this regard, linguistic studies on languages of the Guaicurú family (spoken by Mocovíes, Toba, Pilagás, and Caduveos), typical of the Chaco region and Mato Grosso do Sul, have shown some grammatical morphemes similar to elements of languages belonging to the Macro-Jê linguistic trunk, widely spread throughout the Central and Eastern regions of Brazil [92, 93]. A higher amount of Chaco samples should be analyzed in Y chromosome genomic studies to better understand the human links among these regions.

### Sub-lineage Q-MPB016

The phylogenetic relationship found in this work between an Ecuadorian individual of the Cañari ethnic group and a Brazilian individual of the Hupda ethnic group agrees with that previously described [5]. In the present work we contribute 7 new SNPs shared between both samples, not described in the literature and absent in ISOGG (see S2 Table). The dating found in this study for this sub-lineage is 11.2 kya (9.9–12.7), within the range estimated before [5].

Q-MPB016 provides evidence of a shared lineage ancestry among human groups of the Cañari ethnic group of Ecuador and the Hupda ethnic group of Northwestern Amazonia of Brazil with a temporal depth of 11.2 kya (9.9–12.7). We cannot define whether these groups have a common origin, or if they had different origins and were then linked and admixed leaving shared genetic traits, but genetic evidence of separation of the Cañari lineage from the characteristic lineages of Peru (such as Q-SK281or Q-Z6658) would indicate that the Cañaris managed to preserve their ancestral lineage despite the Inca and Spanish conquest processes. The same is observed for the Hupda ethnic group, which presents a differentiated lineage from those found for neighboring Amazonian ethnic groups such as Arawak (such as Q-Z35497 or Y27993). The links between Cañari and Hupda groups could also be useful for the reconstruction of their ancient history. For a schematic representation of the geographic distribution of Q-MPB016, see S3C Fig.

### Sub-lineage Q-Z5908/Q-B48

Q-Z5908 was found in the present study shared among 6 individuals from Peru, as previously reported [5, 52, 94], 2 individuals from the Province of Salta, Argentina, 1 individual belonging to the Colla ethnic group, and another one from the town of Cachi, also previously described for this lineage [6, 8]. A new Argentine sample in our collection from La Quiaca, Jujuy Province, is added to this sub-lineage as a novelty in this study (S1 Table and S5 Fig).

We have found 69 new SNPs for this lineage, absent in ISOGG (S2 Table), one of which is equivalent for Q-Z5912 and another one is equivalent for Q-Z5910. We have also described a new sub-lineage derived from Q-Z5908 defined by Q-GMP51 (S4 Table and S5 Fig). The remaining 66 SNPs are private for the new sample in our collection, and 7 named Q-GMP52 to Q-GMP58 were validated (see S2 and S4 Tables).

Furthermore, the incorporation of a new high coverage complete sequence to this sub-lineage allows a new estimate of its dating, with values of 13.6 kya (12.0–15.4), older than those reported in the literature [6].

The phylogenetic relationships found for Q-Z5908 show links among human groups from the Central Andes, extending through the territories that today are part of Peru and Northwestern Argentina, with a regional differentiation and defined spatial structure (~13.6 kya). These links resemble what was previously discussed for the Q-B42 lineage in human groups of these regions. For a schematic representation of the geographic distribution of Q-Z5908, see S1B Fig.

### Sub-lineage Q-Z35841

This lineage has been found in the present study in an Argentine individual from the town of Cachi in the province of Salta, as reported in literature [8], and in a Peruvian individual, previously described as part of this lineage [95] (S1 Table and S5 Fig). At present, no study has been able to date it.

The phylogenetic relationship found for this clade between a Peruvian individual and one from Salta provides further evidence to the movements and gene flow observed among human groups of the Central Andes, extending through Northwestern Argentina, reinforcing what was found and discussed for Q-Z5908 and Q-B42. For a schematic representation of the geographic distribution of Q-Z35841, see S4C Fig.

### Sub-lineage Q-Z5906

The Q-Z5906 sub-lineage includes 11 individuals, out of which 5 are Peruvians and 6 are individuals from Northwestern Argentina (S1 Table and S6 Fig). This lineage has been described in the literature as characteristic among members of Peru, Bolivia, Calchaquí communities, and Colla ethnic groups of Argentina [6, 8, 52, 96]. In the present study, 2 new sequences of Argentine individuals are contributed to this sub-lineage, both from La Quiaca, province of Jujuy (see S1 Table and Table A in S1 Text).

We have found 30 new SNPs for this lineage, absent in ISOGG, out of which 8 are equivalent for Q-B35 and 5 were validated as Q-GMP65 to Q-GMP69; downstream, we found a new sub-lineage validated as Q-GMP70, with other 3 SNPs equivalents, 2 of which were validated as Q-GMP71 to Q-GMP72 (see S2 and S4 Tables). We also detected 2 Q-Z5907 equivalents and the remaining 16 were private of the new samples of this lineage (S2 Table).

The gene flow between Andean human groups of Peru and Northwestern Argentina is reflected once again by Q-Z5906 and its sub-lineages Q-B35, Q-GMP70, and Q-Z5907 (S6 Fig), similar to the links found for Q-Z35841, Q-Z5908, and Q-B42 lineages, discussed above for human groups of these regions.

This lineage was found in literature with an estimated dating of 12.88 kya (11.38–14.57) [5]. The present study determined datings of 2.4 kya (2.1–2.7) and 1.7 kya (1.5–1.9) for the derived sub-lineages Q-GMP70 and Q-Z5907respectively (S5 Table). This indicates a great temporal depth for this lineage but with more recent regional differentiation covering the great extension between Peru and Northwestern Argentina, which shows the constant interaction and gene flow of these groups for thousands of years. For a schematic representation of the geographic distribution of Q-Z5906 and its sub-lineages, see S2A Fig.

### Hypothesis of the American settlement

Increasing archaeological evidence proves the early human presence in the American continent. Recent archaeological excavations in the Chiquihuite cave in Northern Mexico demonstrate human occupation dating from ~26,500 years ago [15], and there is even earlier evidence in this country [97]. This evidence joins several documented archaeological sites in Northeastern and Central Brazil that have yielded dates between 20,000 and 30,000 years ago for human occupation in this region [9–13, 98, 99].

**Fig 2:**
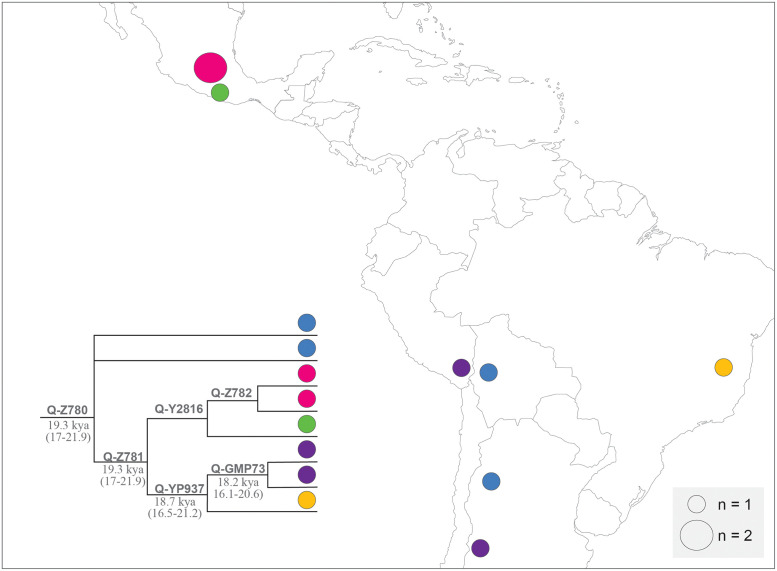
Schematic representation of the geographic distribution of Q-Z780 and sub-lineages. Colored circles represent geographic distribution and sub-lineage membership as shown in the inset tree. Divergence times estimated in this study are represented in italics, in kya, and with a 95% confidence interval between parentheses (for more details see Methods). The size of the circles is related to the number of subjects and is specified with the "n" in the box to the right. Individuals with Mexican ancestry from Los Angeles have been arbitrarily represented in City of Mexico (for more information about samples, see S1 Table). Layer map downloaded from [100].

The current Native American male sub-lineages discussed before in this study are analyzed here according to their temporal depth and are contrasted with updated archaeological information which allows to infer the history of the American settlement. In Fig 2 the geographic distribution of Q-Z780 and its sub-lineages is schematically represented.

Although currently there is practically one single individual from each sub-lineage and geographic area, in the Hypothesis of the American Settlement of this paper, we assume that each individual is representative of the present and past populations of an area in light these are the only data available to date. We acknowledge that a higher number of representatives of these sub-lineages in each area would be very valuable to estimate the frequency and to be able to draw more complete historical inferences, but the extremely low frequency of Q-Z780 makes those representatives quite difficult to be found.

We estimated a temporal depth of 19.3 kya (17–21.9) for Q-Z780 and Q-Z781. This temporal depth and the presence of these lineages in Mexico and Brazil are consistent with the dates estimated for the human presence from the archaeological sites mentioned in Mexico [15] and in Northeastern and Central-Western Brazil [9–13, 98, 99]. As seen in Fig 2, Q-Z781 could be found widely distributed among individuals from Mesoamerica and South America since 19.3 kya (17–21.9). Furthermore, Q-Z781 seems to have undergone a characteristic regional differentiation from Q-Y2816 and Q-Z782 for individuals from Mexico (uncertain dates), Q-YP937 among individuals from Peru, Brazil and Argentina since 18.7 kya (16.5–21.2), and Q-GMP73 among individuals from the Central Andes and Central West of Argentina since 18.2 kya (16.1–20.6). This study provides genetic evidence that supports an early human settlement for Mesoamerica and South America, before 18,000 years ago.

The Younger Dryas (YD) event, spanning 12,900–11,600 cal BP period, was known as a rapid cooling in the Northern hemisphere [23, 101]. Greenland ice core data show a climate reversal from warm to cold at the onset of the YD, and an extremely rapid change back to warm conditions by the end of the YD [102, 103]. There are also records of a fast sea level global rise near the YD period [104]. There is currently no consensus about what caused this event or the exact mechanisms of initiation, stabilization, or termination of the Younger Dryas [24, 105–107].

The YD impact hypothesis states that fragments of a large disintegrating asteroid/comet hit North America, South America, Europe, and Western Asia at 12,800 cal BP [22, 24, 108]. Multiple airbursts/impacts produced the YD boundary layer (YDB, Younger Dryas boundary), depositing peak concentrations of a wide variety of impact markers [24]. The proposed impact event caused major changes in continental drainage patterns, ocean circulation, in temperature and precipitation, large-scale biomass burning, abrupt climate change, abrupt anomalous distribution of plants and animals, extinction of megafauna, as well as, cultural changes and human population decline [22, 24, 109–111]. The diversity of the set of markers related to the cosmic impact is found mainly in the Northern hemisphere [24], including Venezuela [112, 113], but they have also been recorded in the Southern hemisphere, in Chilean Patagonia [22] and Antarctica [114].

The study of how the YD event affected human populations of the time has been considered by archaeologists worldwide [23]. A study carried out to test whether human populations in North America were affected by abrupt climate change or other environmental factors associated with YD, indicates that a significant decline in the human population, or alternatively, the reorganization of populations (that is, drastic changes in settlement patterns), occurred in large areas of North America about 12,900 cal BP. It also shows that similar decreases or changes occurred in much of the rest of the Northern hemisphere, Europe, parts of Asia and Africa [115]. In contrast, the Middle East does not evidence any significant decline in the human population at the beginning of the YD; instead, it shows growth followed by a prolonged plateau, suggesting that the Middle East may have served as a refuge for humans of the time [23, 115, 116].

Unfortunately, there are few archaeological studies that focus on how the YD event affected human populations in Mesoamerica and South America [117–119]. A recent archaeological study reporting how the YD period influenced the human populations of South America, carried out in the Patagonian archaeological site of Pilauco, in Southern Chile [120], shows that lithic artifacts produced by settlers at that time are only found in close association with megafauna remains [22]. This observed connection suggests that humans exploited the extinct megafauna through searching and/or hunting before YDB. On the other hand, no human artifacts were found after the extinction of the megafauna in the YDB layer. This absence of remains could suggest that humans left the area after the extinction of the megafauna and/or experienced a decrease and/or reorganization of the regional population [22], as proposed for North America [115]. Furthermore, the studies carried out in Pilauco to infer the local botanical history before and after YD found a significant decrease in the number and diversity of plant materials and a marked change in the vegetation taxonomic composition [22].

Currently, analyses of the effects caused at the level of the human genome by the environmental changes produced during the YD period are rather scarce. There is increasing evidence of large-scale biomass burning during the YD recorded in Europe, Asia, and North America, and even South America, where the archaeological record is consistent with regional forest fire activity, which indicates that anomalous biomass burning reached high latitudes in the Southern hemisphere [22, 112]. These environmental alterations could potentially have affected human populations genomes at the time in a number of ways: the extinction of primary food sources could have drastically altered their diet, perhaps exposing them to new mutagens for which humans had not yet evolved to avoid or metabolize [121, 122]. Moreover, particulate matter from biomass burning has recently been related to various forms of DNA damage both *in vivo* and *in vitro* [123, 124].

In this work it is proposed that the population foci that existed ~19.3 kya in what is now Mexico, Peru, Argentina, and Brazil could have been drastically affected by the environmental episode that occurred during the YD period. The Q-Z780 lineage, and perhaps Q-F4674, could have comprised a much larger population before the YD, and suffered after this event a substantial decrease that could not be reverted to the number it showed previously to the YD. Therefore, we propose that the environmental events occurred during the YD could represent one of the fundamental reasons for the current low frequency of the Q-Z780 and Q-F4674 lineages in the Americas.

The most prevalent Native American lineage currently Q-M848, with a temporal depth of ~15.4 kya, coexisted before the YD event along with Q-Z780, but seems to have been more successful in surviving to the environmental changes that occurred during the YD period. If we consider the divergence times average, only 3 Q-M848 sub-lineages are older than 12.9 kya (see S1 Fig), and all the remaining fall within the YD period or later (see S2–S4 Figs). Surprisingly, the upper and lower limits of all divergence times of Q-M848 sub-lineages found fall within the time period encompassed by the YD event or later. The environmental events that occurred during the YD period could have acted as a driving force for the expansion and diversification of Q-M848 sub-lineages, generating the star-shaped topology of Q-M848 described above [7], with a spatial structure that developed during the YD period.

Most of the genetic records of current male Native American sub-lineages are those derived from Q-M848 after 12,900 cal BP, so, much of the current genetic diversity present in male Native American lineages could be represented by those sub-lineages that achieved to survive, expand and diversify after the environmental events of the YD period. This could suggest an extinction and loss in the gene pool of a large part of the sub-lineages existing previously to the environmental events during this period.

In this regard, a recent archaeological study compiles the data set of 1,661 radiocarbon early dates (1,543 made on cultural materials or related remains and 118 on human bones/teeth) from 454 archaeological sites in South America (including only archaeological sites after 15,000 cal BP meeting certain standard site validation requirements) [4]; it was screened for early demographic change using summed probabilities and showed that during 15,100–13,500 cal BP the intensity of the archaeological signal was extremely low (the archaeological intensity signal peaks at ~12,500 cal BP). These results are similar to those explained above about the dating of the most frequent Native American sub-lineage Q-M848 and could represent those human groups that managed to survive the environmental adversities in the YD period.

Prates et al. [4] also suggest that if a pre-15,500 cal BP first arrival in South America indeed occurred, this early population would have probably become extinct, since alleged cultural evidence before this date shows a substantial (and unexpected) discontinuity, and human remains are completely absent before 12,600 cal BP. In the present study, we found a significant temporal discontinuity between the spatial structure of Q-Z780 and Q-M848 sub-lineages. The Q-Z780 and Q-F4674 lineages could be part of the extremely low archaeological signal before 15,500 cal BP, that was near-extinction but managed to survive the environmental events during YD period and is preserved among modern Native American lineages at low frequencies. It is highly probable that the absence of human remains before 12,600 cal BP observed [4] is related to the environmental events that occurred during the YD, which leads us to wonder: what relationship could the absence of human remains before 12,600 cal BP have with large-scale biomass burning in South America? [22, 112]. What other records and biological evidence prior to 12,900 cal BP could have been lost in this event?

Considering an abrupt population decline in Native American populations as a result of the environmental adversities during the YD period makes it more difficult to understand the demographic processes that underlie the current distribution of the Q-M242 haplogroup in Euro-Asia and its links with the Native American lineages. We propose that if the Middle East did not have a population decline during the YD period but rather served as a refuge for humans of the time [23, 115, 116], the Q Haplogroup lineages older than ~12,900 years (such as Q-L275) could have been preserved to a greater extent during the YD event in the Middle East region, and after this event, disperse and differentiate until reaching the diversity existing today for Q Haplogroup in Euro-Asia. Given the lack of genetic data about ancient Q-M242 sub-lineages (before 12,900 cal BP) in the Americas, further studies are still required to estimate their place of origin since it could be an overestimation to define an Asian origin for Q-M242.

Another important aspect for the study of the history of the American settlement is that global sea levels rose sharply and rapidly by ~120 m by the time of the YD [104]. In South America, early submerged prehistoric sites are virtually unknown. Recent investigations in the Pacific coast of Central Chile revealed that ~16,000 years ago, after a dramatic sea level lowering, a significant part of territories remained exposed and available for terrestrial fauna. A great diversity of submerged extinct terrestrial megafauna was found there [125]. This evidence suggests that favorable conditions and resources existed for human groups ~16,000 years ago or earlier in territories that could have served as sites for human settlement and/or used as migratory routes but that are now under water; thus, we still observe a great bias in terms of territorial data that would also be important to analyze for the historical reconstruction of the American settlement.

## Conclusions

Q-Z780 and Q-Z781 sub-lineages, autochthonous of the Americas, presented a wide distribution in Mesoamerica and South America since ~19,300 years ago. This contributed to a regional differentiation from Q-Y2816 and Q-Z782 for individuals from Mexico, Q-YP937 among individuals from Peru, Brazil, and Argentina since ~18,700 years, and Q-GMP73 among individuals from the Central Andes and Central West of Argentina since ~18,200 years ago. Moreover, it provided genetic support for South American settlement before 18,000 years ago, in agreement with a long chronology scenario and the dates of Meso- and South American archaeological sites [9–13, 15, 98, 99].

The Q-Z780 lineage, and perhaps Q-F4674, could have suffered a substantial drop due to the environmental events occurred during the YD, which could be the main reason for its current low frequency. For the Q-M848 lineage the YD environmental events could have acted as a driving force for its expansion and diversification, and while they could have also caused a substantial decrease, this lineage survived more successfully than Q-Z780. The upper and lower limits of all divergence times for Q-M848 sub-lineages cover the YD period or later. The spatial structure of the South American male population at ~12.3 kya [5], and the archaeological intensity signal peak at ~12,500 cal BP [4] could represent those human groups that managed to survive the environmental events occurred during the YD period (~12,800 years ago), in full process of expansion and diversification.

Understanding the relationship between the Eurasian and American Q-M242 lineages becomes more complex when population changes during the YD period are included [23, 115]. Since there was no population decline in the Middle East during the YD period but it rather served as a refuge for humans of the time [23, 115, 116], then, Q Haplogroup lineages prior to ~12,900 years ago (such as Q-L275) could have been preserved to a greater extent during the YD event in the Middle East region and after this disperse and differentiate until reaching the diversity that exists today for Q Haplogroup in Euro-Asia. However, Q Haplogroup Native American sub-lineages older than ~12,900 years ago would have suffered a drastic decline in the YD period, altering the ancient spatial structure (before 12,900 cal BP) of the human populations of the Americas and causing the extinction of lineages and loss of part of the gene pool. Further studies should include ancient Native American sub-lineages (before 12,900 cal BP) in order to understand and estimate the origin of Q-M242.

The reconstruction of the first South American settlements still requires further studies. Y chromosome NGS sequencing has proven to be a highly important tool, and future studies with a larger number of diverse individuals with Native American ancestry from all over the continent, as well as a larger number of sequences belonging to ancient Native American sub-lineages, would highly contribute to this field. Furthermore, in order to gain insight into our ancient history, additional multidisciplinary efforts should focus on studying what caused the environmental changes that occurred during the YD period and what the spatial structure of the environment and human populations were like before this event.

## Methods

### Sampling

Argentine samples were collected from volunteers from San Juan (San Juan), Malargüe (Mendoza), Lavalle (Mendoza), La Quiaca (Jujuy), Santa María (Catamarca), Belén (Catamarca), Tartagal (Salta), Bariloche (Rio Negro), and El Chalía (Chubut) during campaigns carried out at different times. Samples were evaluated by two different Ethics Committees: Provincial Bioethics Committee of the Province of Jujuy and Instituto Multidisciplinario de Biología Celular (IMBICE) Ethics Committee [25]. All biological samples are accompanied by a genealogical survey in which the volunteer reveals his place of birth, the place of birth of his maternal lineage and his paternal lineage (Paternal Origin), as well as his socio-ethnic origin (if they have knowledge of Native American or foreign ancestry), and his knowledge or not of any language other than Spanish. These data allow disregard related people to avoid duplicate lineages. The DNA samples were extracted from 15 ml of blood through the “salting out” method [139]. Haplogroup determination was made by Polymerase Chain Reaction-Product Length Polymorphism (PCR-APLP) designed by Umetsu et al. [140, 141] and adapted according to Jurado-Medina et al. [142]. Q Haplogroup was identified in individuals using specific primers for Q-M242, Q-M3, and Q-M346 markers following the multiplex PCR-APLP technique [142], and Q-Z780 and Q-Z19231 through sequencing markers [38, 142]. We selected the samples belonging to the Q-M3 and Q-Z780 haplogroups according to their haplotypes in a network built with 17 STR (YFiler) previously reported [25]. The sample belonging to Q-M346* was selected to obtain more information about this minority haplogroup [38]. For more information about selected samples, see Table A in S1 Text and S1 Table.

### Genomic sequence data generation and NGS data processing

The DNA from the 13 samples was sent to Full Genomes Corporation [143] for sequencing of the whole Y chromosome with Y Elite 2.1 service. Illumina HiSeq 4000 equipment was used which offers paired-end runs with 150 bp length of reads, 30x coverage, and Agilent SureSelect target enrichment system. We received the results in Fastq format.

Paired-end reads obtained from sequencing of whole Y genomes samples were analyzed using FastQC [129] and trimmed for low quality and GC bias using Prinseq [130]. Then they were aligned to the GRCh37 reference human genome using the Burrows-Wheeler Alignment Tool (BWA) [131]. The resulting SAM files were filtered to keep only the Y chromosome section [132], duplicated reads were removed, and the quality scores recalibrated (using 1000 Genomes phase1indels and high confidence SNPs [144]) by the implementation of the Genome Analysis Toolkit (GATK) framework [133]. For a summary of the software used, see Table 1. For more information about NGS processing of the new sequences obtained, see S1 Text.

**Table 1:** Software summary table.

FastQC v. 0.11.8 [129]	https://www.bioinformatics.babraham.ac.uk/projects/fastqc/
Prinseq-lite v. 0.20.4 [130]	http://prinseq.sourceforge.net/
Burrows-Wheeler Alignment Tool (BWA) v. 0.7.17 [131]	http://bio-bwa.sourceforge.net/
SAMtools v. 1.7–1 [132]	http://samtools.sourceforge.net/
Genome Analysis Toolkit (GATK) v. 3.8.1 [133]	https://software.broadinstitute.org/gatk/
VCFkit v. 0.1.6 [134]	https://vcf-kit.readthedocs.io/en/latest/
VCFtools v. 0.1.15 [135]	http://vcftools.sourceforge.net/
BCFtools v. 1.7–2 [136]	https://samtools.github.io/bcftools/bcftools.html
RAxML v.8.2.12 [137]	https://cme.h-its.org/exelixis/software.html
FigTree [138]	http://tree.bio.ed.ac.uk/software/figtree/

We reviewed public databases for more samples belonging to the Q-M242 haplogroup (see Table 2). Some were available with complete sequence data in BAM format (see Table B in S1 Text), while others were available from VCF format (described in Table D in S1 Text). This difference in the samples available from BAM and in VCF led us to work separately on two groups of samples. With the set of sequences in BAM format; including the new ones from this study, intermediate GVCFs were created using the HaplotypeCaller parameters in ploidy 1 from GATK. Joint genotyping of multiple samples was then performed using GenotypeGVCFs and StandardAnnotation from GATK. Once the joint GVCF was created, a series of data filter processing was performed, including the elimination of repetitive regions of the Y chromosome, indels, and missing data or "missingness" higher than 0.1 (Table C in S1 Text). With the other group of samples, sequences available in VCF format were joined into a unique file with "CombineVariants" from GATK, and then the same series of data filter processing explained above for the sequences in BAM format was applied (see Section 3 and Table E in S1 Text). Then, all the sequences of this study were assembled by applying CombineVariants and UNIQUIFY parameters of the GATK platform; in this file the monomorphic alleles were filtered and all the positions that had a reading depth lower than 2 were filtered (see Section 4 and Table F in S1 Text).

**Table 2:** Sequence data summary table.

Q Haplogroup Y chromosome sequence data	Source
13 Argentine	This study
18 Native American	Pinotti et al. [5]
11 Native American	Karmin et al. [8]
12 Euro Asian and Native American	The Simons Genome Diversity Project [126]
45 Euro Asian and Native American	1000 Genomes Consortium [127]
4 Northern European	J Norstedt; A Solli; K Dawtry; K Reed & Q Nordic Family Tree DNA group project [128]

### Phylogeny and dating

We built a phylogenetic tree from 102 sequences belonging to Q-M242 haplogroup plus a sequence from B2b1 haplogroup (from the Democratic Republic of Congo, Africa) used as a phylogenetic root. A multiple sequence alignment (MSA) was performed using the "phylo fasta" option of the VCF-kit software [134, 145], the generated FASTA file only incorporates the variable sites from VCF samples.

For the construction of the maximum likelihood phylogenetic tree, the parameters of Pinotti et al. [5] were followed. We used the Randomized Axelerated Maximum Likelihood software (RAxML) v.8.2.12 [137, 146] in three steps: first, a "best tree" was generated, in the next step a bootstrap analysis with 100 re-samples was performed, and finally, a "consensus" tree was generated using the best tree and bootstrap results.

For the dating of the phylogenetic nodes, the calculation of ρ (rho) statistics [147] was used following the same methodology used by Pinotti et al. [5]. The Y chromosome mutation rate reported by Fu et al. [148] of 0.76x10^-9^ mutations per site per year was used, with a 95% confidence interval of 0.67x10^-9^ to 0.86x10^-9^.

Since dating calculations are affected by the amount of information from the sequences, in this study for the dating estimates we used only those samples that are available as complete sequences (see section 2 in S1 Text), which were also filtered under the same criteria, and thus we assumed a balance between the number of variant and invariant sites. This allowed us to estimate only the dating of nodes consisting of high coverage complete sequences. All the dates estimated in this study are presented in Fig 1. Since the dates of nodes composed of available sequences in VCF (see section 3 in S1 Text) obtained from the literature are used in the discussion section, we have also included these dates in S5 Fig for a better reading and understanding of this work; for a complete list of the sources of all dates used in this figure, see S5 Table.

### Search for variants of phylogenetic importance and validation of new SNPs

Taking into account the clades of the generated consensus phylogenetic tree and in order to find the variant sites present in these, using the "SelectVariants" parameter of GATK, a VCF file was created for each clade that included only the samples of the clade to be analyzed, and on the other hand a VCF file containing all the samples of the tree excluding the samples of the clade to be analyzed. Subsequently, with the BCFtools "isec" tool, a file was generated containing the intersection between the two generated VCFs. This file contained all the variants of the clade under study. A manual search was carried out in the SNPs databases of ISOGG [40, 149] as well as in the last works published on the subject [5–8, 150] in order to know if the variants found in this study had been validated. For the selection of SNPs to be validated by Sanger method, we used first those shared among several samples of a sub-lineage that were not validated by other authors and absent in ISOGG; we also chose private variants of new samples sequenced in this study. For a complete list of new variants found in this work, see S2 Table. For more information about the experimental conditions and the designed primers, see section 5 in S1 Text, S3 and S4 Tables.

## Supporting information

S1 Text Additional information. (DOCX)

S1 Fig Geographical distribution of the sub-lineages Q-MPB139 (A), Q-Z5908 (B), and Q-B42 (C). Colored circles represent the geographic distribution and sub-lineage membership, as shown in the inset tree. The size of the circles is related to the number of subjects and is specified with the "n" in the box to the right, see S1 Table. Estimated sub-lineage divergence times are represented in italics, in kya, and with a 95% confidence interval between parentheses (for more details see Methods), those shown without asterisks are dates estimated in this study, those with an asterisk are taken from the literature, see S5 Table. Nodes that do not present dates are those that could not yet be estimated. Dotted lines indicate that their phylogenetic link still needs to be further studied and confirmed. Layer map downloaded from [100].
(PNG)

S2 Fig Geographical distribution of the sub-lineages Q-Z5906 (A), Q-Y27993 (B), and Q-SK281 (C). Colored circles represent the geographic distribution and sub-lineage membership, as shown in the inset tree. The size of the circles is related to the number of subjects and is specified with the "n" in the box to the right, see S1 Table. Estimated sub-lineage divergence times are represented in italics, in kya, and with a 95% confidence interval between parentheses (for more details see Methods), those shown without asterisks are dates estimated in this study, those with an asterisk are taken from the literature, see S5 Table. Nodes that do not present dates are those that could not yet be estimated. Dotted lines indicate that their phylogenetic link still needs to be further studied and confirmed. Individuals with Mexican ancestry from Los Angeles have been arbitrarily represented in City of Mexico (S1 Table). Layer map downloaded from [100].
(PNG)

S3 Fig Geographical distribution of the sub-lineages Q-CTS2731 (A), Q-CTS11357 (B), and Q-MPB016 (C). Colored circles represent the geographic distribution and sub-lineage membership, as shown in the inset tree. The size of the circles is related to the number of subjects and is specified with the "n" in the box to the right, see S1 Table. Estimated sub-lineage divergence times are represented in italics, in kya, and with a 95% confidence interval between parentheses (for more details see Methods), those shown without asterisks are dates estimated in this study, those with an asterisk are taken from the literature, see S5 Table. Nodes that do not present dates are those that could not yet be estimated. Dotted lines indicate that their phylogenetic link still needs to be further studied and confirmed. Individuals with Mexican ancestry from Los Angeles have been arbitrarily represented in City of Mexico (S1 Table). Layer map downloaded from [100].
(PNG)

S4 Fig Geographical distribution of the sub-lineages Q-Z19357 (A), Q-MPB118 (B), and Q-Z35841 (C). Colored circles represent the geographic distribution and sub-lineage membership, as shown in the inset tree. The size of the circles is related to the number of subjects and is specified with the "n" in the box to the right, see S1 Table. Estimated sub-lineage divergence times are represented in italics, in kya, and with a 95% confidence interval between parentheses (for more details see Methods), those shown without asterisks are dates estimated in this study, those with an asterisk are taken from the literature, see S5 Table. Nodes that do not present dates are those that could not yet be estimated. Dotted lines indicate that their phylogenetic link still needs to be further studied and confirmed. Layer map downloaded from [100].
(PNG)

S5 Fig Detailed phylogeny of Q-M242 haplogroup constructed with 102 Y chromosome sequences. The colors of each individual of the tree are according to the macro-area or the country according to the box on the left. The dashed lines are used to represent branches that require to be further studied for a better definition. The length of the branches is represented as a function of time in kya according to the axis represented. The nodes without asterisks are those dated in this study. The red asterisks are the nodes that could not be dated and the length of their branches does not represent a defined time depth. The green asterisks represent the dates taken from the literature; for a complete list of the dates used in this figure, see S5 Table. For more information about data set, see S1 Table and Table A in S1 Text.
(PDF)

S6 Fig Number of SNPs found for each node and branch of the reconstructed Q-M242 phylogenetic tree. The length of the branches is expressed as a function of the number of SNPs found in each one. The asterisk indicates samples available only as VCF (see Section 3 in S1 Text and S1 Table).
(PDF)

S1 Table Samples information. (XLSX)

S2 Table New SNPs found. (XLSX)

S3 Table Sequence of primers and conditions of each pair. (XLSX)

S4 Table New SNPs validated and not validated. (XLSX)

S5 Table Dating used for the construction of the calibrated phylogenetic tree in S5 Fig. (XLSX)
