# Supplementary material for: Human Y chromosome sequences from Q Haplogroup reveal a South American settlement pre-18,000 years ago and a profound genomic impact during the Younger Dryas
Source: PLoS One. 2022 Aug 17;17(8):e0271971. doi: 10.1371/journal.pone.0271971 (PMC9385064; doi:10.1371/journal.pone.0271971)
Supplement: S1 Text — (DOCX) [file pone.0271971.s001.docx]

Supplementary text

Table A. Summary of Samples Selected for NGS Sequencing.

| **Sequencing ID-Code** | **Internal code** | **SNP** | **Haplogroup** | **Location in Argentina** | **Source** |
| --- | --- | --- | --- | --- | --- |
| N8A2QN | B203 | M3 | Q1b1a1a | Bariloche (Rio Negro) | This study |
| LD4PC | LQ60 | M3 | Q1b1a1a | La Quiaca (Jujuy) | Jurado Medina et al. [1] |
| N87FK8 | TG71 | M3 | Q1b1a1a | Tartagal (Salta) | Jurado Medina et al. [2] |
| Z8ZMY | Be89 | Z780 | Q1b1a2 | Belén (Catamarca) | Jurado Medina et al. [3] |
| SQVCW | TG33 | M3 | Q1b1a1a | Tartagal (Salta) | Jurado Medina et al. [2] |
| TYEQC | SMA138 | M3 | Q1b1a1a | Santa María (Catamarca) | Jurado Medina et al. [1] |
| T4WQV | LQ41 | M3 | Q1b1a1a | La Quiaca (Jujuy) | Jurado Medina et al. [1] |
| EKEFB | LQ6 | M3 | Q1b1a1a | La Quiaca (Jujuy) | Jurado Medina et al. [1] |
| RUTBE | SJN49 | M346 | Q1b | San Juan (San Juan) | Jurado Medina et al. [3] |
| M39DJ | LV59 | M3 | Q1b1a1a | Lavalle (Mendoza) | Jurado Medina et al. [1] |
| 6QHWE | SMA111 | M3 | Q1b1a1a | Santa María (Catamarca) | Jurado Medina et al. [1] |
| UCNEN | TEH26 | M3 | Q1b1a1a | El Chalía (Chubut) | This study |
| S8BAL | MLG132 | Z780 | Q1b1a2 | Malargüe (Mendoza) | Jurado Medina et al. [3] |

Detail of the 13 samples selected for complete Y chromosome NGS sequencing.

1. NGS data processing of the 13 sequenced samples

The NGS sequencing data processing was performed using the Best Practices Recommendations of GATK (Genome Analysis Toolkit) version 3.8.1 [4, 5]. Following these recommendations, we developed an executable script with the pipeline for processing NGS data from the 13 samples sequenced in this work. This pipeline contains multiple commands, including the processing of the sequences from the raw ".fastq" file to the generation of an "intermediate" GVCF. The pipeline was run for each file obtained from the sequencing (13 x 2 .fastq files in total, since there are 13 sequences with paired-end results). The steps in this workflow will be explained below, using the sample named LD4PC as an example.

1.1 Quality control of sequencing

The quality control process of the sequencing was carried out with the FastQC software [6]. This program allows raw data in .fastq format to undergo quality control of raw sequence data that are verified before any further analysis is performed.

The FastQC software (Version 0.11.8) was downloaded from the Babraham Bioinformatics platform [7], it requires a suitable Java environment and the installation of the Picard library, which is included in the download. The command used for each .fastq file was:

**fastqc LD4PC_HNHYNCCXX_L6_1.clean.fq LD4PC_HNHYNCCXX_L6_2.clean.fq –extract**

1.2 Low-quality sequencing data filters

The PRINSEQ software [8] was used as a tool to filter, reformat and clip NGS sequencing data with low sequencing quality. The prinseq-lite 0.20.4 program was downloaded from [9]. The command used was:

**perl prinseq-lite.pl -fastq LD4PC_HNHYNCCXX_L6_1.clean.fq -fastq2 LD4PC_HNHYNCCXX_L6_2.clean.fq -out_good LD4PC_ QC -min_qual_mean 28 -trim_left 10 -trim_right 10 -log LD4PC_prinseq.log**

1.3 Alignment and mapping against the reference human genome

Once the sequence reads were filtered and we obtained those with a minimum quality threshold, the alignment and mapping step was continued where the sequences were located in order to reconstruct the complete sequence of the amplified DNA. This processing was carried out using the human genome reference version 37 (Genome Reference Consortium Human Build 37, GRCh37), obtained in ".fasta" format from [10]. The software used was Burrows-Wheeler Aligner (BWA) [11, 12]; we used the BWA-MEM algorithm designed for short sequences, between 35 and 300 bp. The command used was:

**bwa mem -M -t 9 human_g1k_v37_decoy.fasta LD4PC_QC_1.fastq LD4PC_QC_2.fastq > LD4PC _bwa_mem.sam > LD4PC _bwa_mem.log**

1.4 Aligned and mapped file ordering

To sort the SAM file generated in the previous step, we used the Picard software [13], which is a Java toolkit that presents the SortSam function, which allows ordering the SAM file and converting it to its binary version in BAM. The command used was:

**java -jar picard.jar SortSam I=LD4PC_bwa_mem.sam O=LD4PC.sorted.bam SO=coordinate CREATE_INDEX=true > LD4PC.sortsam.log**

1.5 BAM file processing

The next steps were a series of processings performed on the BAM files. The general mapping statistics, such as the number of reads that aligned correctly against the reference, were first checked with Samtools' "flagstat" tool [14, 15]. The command used was:

**samtools flagstat LD4PC.sorted.bam > LD4PC.flagstat_before.stats**

In the next step, using the Samtools "view" parameter next to "REGIONS", only the alignments assigned to the reference sequence of the Y chromosome were printed. The command used was:

**samtools view -bh -F 256 -f 2 LD4PC.sorted.bam $REGIONS Y > LD4PC.chrY.bam**

After printing only the Y chromosome reads, we saw the mapping statistics for the Y chromosome only, which was what would be ultimately used in the analysis:

**samtools flagstat LD4PC.chrY.bam > LD4PC.flagstat_after.stats**

Duplicates were identified and flagged with a "FLAG" in the BAM files with the Picard program [13] so that the variant calling programs (later steps) would not take them into account when establishing the genotypes for each variant. The command used was:

**java -jar picard.jar MarkDuplicates I=LD4PC.sorted.bam O=LD4PC.dedupped.bam M=LD4PC.dedupped.metrics.txt > LD4PC.markduplicates.log**

Then we added metadata from the sequencing experiment to the reads using GATK AddOrReplaceReadGroups; the command used was:

**java -jar picard.jar AddOrReplaceReadGroups I=LD4PC.dedupped.bam O=LD4PC.RG.bam LB=library PL=Illumina SM=LD4PC CREATE_INDEX=true > LDP4PC.addorreplacereadgroups.log**

Base Quality Score Recalibration (BQSR) was performed as a data pre-processing step to detect systematic errors made by the sequencing equipment when estimating the precision of each base call. To carry out this step we used the GATK "BaseRecalibrator" function that generates a model of the covariation of the data provided with a set of known variants. The set of known variants was downloaded from the databases of the Project 1000 Genomes phase I, both indels as SNPs [16]. The command used was:

**java -jar GenomeAnalysisTK.jar -nct 4 -T BaseRecalibrator -I LD4PC.RG.bam -R human_g1k_v37_decoy.fasta -knownSites 1000G_phase1.indels.b37.vcf -knownSites Mills_and_1000G_gold_standard.indels.b37.vcf -knownSites dbsnp_137.b37.vcf -o LD4PC.recal.table > LD4PC.baserecalibrator.log**

The next step was "print reads", in which the quality of the bases in the sample is adjusted according to the model created from the previous step, BQSR (Base Quality Score Recalibration). A filter called well-formed read filter (WellformedReadFilter) was applied which checks if a reading is "well formed", that is, if it is free of large internal inconsistencies and problems that could lead to later errors. Only the reads that pass the filter are "printed" (PrintReads), creating a new BAM file. For this step we also used the GATK platform; the command used was:

**java -jar GenomeAnalysisTK.jar -nct 4 -T PrintReads -R human_g1k_v37_decoy.fasta -I LD4PC.RG.bam -BQSR LD4PC.recal.table -o LD4PC.recal.bam > LD4PC.printreads.log**

1.6 Generation of intermediate GVF prior to calling variants

For the variant calling process, we used GATK's “HaplotypeCaller” tool following the Broad Institute's recommended practices [17, 18]. Since this work aims at building a phylogenetic tree, it is necessary to call the variants for all the samples together, on the one hand because GATK uses the information from all the samples to determine the variants (for example, shared variations between samples are probably considered true variants) and, on the other hand to be able to compare variants among samples. To do this, we worked with a type of VCF that contains additional information, called genomic VCF or GVCF (Genomic Variant Call Format), which is a multi-sample VCF which contains information on all the variants of all the samples.

The construction of the GVCF is carried out by first executing the HaplotypeCaller tool sample by sample to generate an intermediate GVCF (which will not be used in the final analysis); then the "GenotypeGVCF" tool is used to generate a joint genotyping of multiple samples in a very efficient way. The command used was:

**java -jar GenomeAnalysisTK.jar -R human_g1k_v37_decoy.fasta -T HaplotypeCaller -L Y -I LD4PC.recal.bam -o LD4PC.haplotypecallerGVCF.g.vcf --emitRefConfidence BP_RESOLUTION -ploidy 1 > LD4PC.haplotypecallerGVCF.log**

The pipeline generated for the 13 samples sequenced in this study ended in this step with the creation of 13 intermediate GVCF files.

2. Assembly of samples available as complete sequences from NGS databases and data processing

The current definition of the phylogeny of Q-M242 haplogroup is low, and this is attributed to the low number of sequenced samples for this haplogroup. In this work, a search was carried out in public access databases for Y-chromosome sequences belonging to the Q-M242 haplogroup, in order to build a phylogenetic tree including as many sequences as possible from this haplogroup. Some of the samples are available as complete sequences in BAM format, while others are not available as complete sequences and are in VCF format, this makes NGS data processing different for samples available in one or another format, in this section the data processing for the first case will be detailed.

The Simons Genome Diversity Project (SGDP) has carried out the complete genomic sequencing of more than one hundred diverse human populations; we included some sequences of the Q Haplogroup of this project in this study, along with other high coverage complete sequences presented by other authors that are detailed in Table B. A sample belonging to B1b1 haplogroup was included for a correct rooting of the phylogenetic tree.

**Table B. Complete sequences available in BAM format downloaded from databases.**

| Complete sequences of the Y Chromosome in BAM format. | Source |
| --- | --- |
| 18 Q-M242 haplogroup sequences | Pinotti et al. 2019 [19] |
| 11 Q-M242 haplogroup sequences | SGDP [20] |
| 4 Nordic sequences of Q-M242 haplogroup | J. Norstedt & Group [21] |
| 1 B1b1 haplogroup sequence for phylogenetic root | SGDP [20] |
|  |  |

2.1 Index generation

In order to assemble the 13 samples sequenced in the present work with the 34 samples downloaded from the databases (in BAM format) and to call the variants together, we first generated the "indexes" (necessary for the processing of the BAMs) for each one of them, using the Samtools platform [15, 22]. Therefore, for each of the 34 samples from the databases, the command used was:

**samtools index Nmuestra.sorted.bam**

2.2 Generation of intermediate GVCF

The intermediate GVCF was then generated for each of the 34 samples. Therefore, the command in section 1.6 was run, generating 34 intermediate GVCF files.

2.3 Genotyping of Multiple Samples

A joint variant call was made running the command:

**java -jar GenomeAnalysisTK.jar -R human_g1k_v37_decoy.fasta -T GenotypeGVCFs -G StandardAnnotation -o set_of_44_samples.genotypeGVCF.vcf -allSites --variant sampleN1.haplotypecallerGVCF.g.vcf --variant sampleN2.haplotypecallerGVCF.g.vcf -- variant sampleN44.haplotypecallerGVCF.g.vcf > set_of_44_samples. genotypeGVCF.log 2>&1**

The result was a single "raw" VCF file that featured a total of 59.371.951 variant sites.

2.4 Variant filtering in complete sequences

To build a robust and calibrated phylogenetic tree a series of additional filtering steps were followed to "clean" the generated "raw" VCF. The filter parameters established in this work were set following both the recommendations of good genomic practices of GATK (Best Practices) [5] and those referring to the manipulation of genomic sequences of the Y chromosome [23] [24] [25].

2.5 Elimination of highly repetitive regions of the Y Chromosome

The Y chromosome has a total length of approximately 60 Mb, but only in a fragment of a length of approximately 10 Mb can unique sites be unambiguously described. Therefore, here we selected only these unique regions for the search for informative phylogenetic variants, following the recommendations and using the region ".bed" file from [24] which presents the unique coordinates of the Y chromosome. The command used was:

**java -jar GenomeAnalysisTK.jar -T SelectVariants -R human_g1k_v37_decoy.fasta --variant set_of_44_samples.genotypeGVCF.vcf -o REG.genotypeGVCF.vcf -L REGY.bed > REG.genotypeGVCF.log**

The generated VCF file has a total of 10.445.027 variant sites.

2.5.1 Deletion of indels

The phylogenetic tree built was based on biallelic polymorphisms (SNPs), so we excluded all indels in this step; the running command was:

**java -jar GenomeAnalysisTK.jar -T SelectVariants -R human_g1k_v37_decoy.fasta --variant REG.genotypeGVCF.vcf --selectTypeToExclude INDEL -o noindels.genotypeGVCF.vcf > noindels.genotypeGVCF.log**

The resulting file contains a total of 10.442.285 variant sites.

In this step it is necessary to rigorously check the absence of all the indels of the generated VCF file. As some had not been removed in the previous step, they were manually found using linux "nano", "awk" and "sed" tools, and then annotated in a ".txt" file which presents a single column of positions. They were finally removed using the VCFtools software version 0.1.15 [26], which provides a set of tools for manipulating VCF files. The command ran was:

**vcftools --vcf noindels.genotypeGVCF.vcf --exclude-positions indels_extras.txt --out noindels2.genotypeGVCF --recode**

The resulting VCF file has a total of 10.441.434 variant sites.

2.5.2 Removal of "missingness" variants

The so-called "missingness" variants appear due to the lack of sequencing coverage in some chromosomal regions. In practice, variants that present a frequency of missing data higher than 0.1 [23] are eliminated.

To do this, a report is first generated of all the sites that present missing variants with their respective frequencies, using the "missing-site" parameter of VCFtools.

**vcftools --vcf noindels2.genotypeGVCF.recode.vcf --** **VCFTools -missing-site**

This "out.lmiss" file is manually ordered from highest to lowest according to the frequency column and a new text file is generated that presents a single column with the positions of frequency higher than 0.1, showing the positions that we want to remove. Then with VCFtools we run the command:

**vcftools --vcf noindels2.genotypeGVCF.recode.vcf --exclude-positions miss_0.1.txt --out alta_cobertura_limpio --recode**

The resulting file has 7.643.332 variant sites.

Elimination of repeating regions (GATK SelectVariants, ChrY Unique Regions)

**Table C. Summary of filters applied in complete sequences.**

|  | **Number of variants conserved per filter applied** |
| --- | --- |
| Elimination of repeating regions (GATK SelectVariants, ChrY Unique Regions) | 10.445.027 |
| Elimination of indels (GATK -selectTypeToExclude INDEL) | 10.442.285 |
| Manual elimination of indels (VCFTools exclude-positions) | 10.441.434 |
| Elimination of "missigness" variants > 0.1 (VCFTools -missing-site) | 7.643.332 |

3. NGS data processing of sequences obtained from databases available in VCF format

This section details the data processing performed to assemble the available Q-M242 sequences in VCF format downloaded from the databases. Table D details the number of streams downloaded and the source.**Table D. Sequences available in VCF downloaded from database.**

| **Sequences of Q-M242 haplogroup** | **Source** |
| --- | --- |
| 45 Y chromosome sequences | 1000 Genomes Project [16] |
| 11 Y chromosome sequences | M. Karmin et al. [27] |

3.1 Union of sequences available in VCF format

The first thing to do was merging them into a single 56-sample file for processing. For this, the "CombineVariants" tool of the GATK platform was used, which allows generating a single VCF file from several samples from different workflows. The following command was run:

**java -jar GenomeAnalysisTK.jar -T CombineVariants -R human_g1k_v37_decoy.fasta --variant cada_muestra_baja_cobertura.vcf -o 1000g_karmin.vcf -genotypeMergeOptions UNIQUIFY**

The generated file presents a total of 56 samples and 22.802.845 variant sites.

3.2 Variants filtering in sequences available in VCF format

A series of variants filterings were performed; these steps were the same as those described in section 2.4; here we only present the summary of filters applied and the record of the number of variants conserved in each step that are shown in Table E.

**Table E. Summary of filters applied in sequences available in VCF.**

|  | **Number of variants conserved per filter applied** |
| --- | --- |
| Elimination of repeating regions (GATK SelectVariants, ChrY Unique Regions) | 10445971 |
| Elimination of indels (GATK -selectTypeToExclude INDEL) | 10444021 |
| Manual elimination of indels (VCFTools exclude-positions) | 10443805 |
| Elimination of "missingness" variants > 0.1 (VCFTools -missing-site) | 60021 |

4. Assembly and processing of the set of all the sequences in this study

4.1 Merging of all samples

Once the available stream files in BAM and VCF were "clean" of most of the spurious variants, the two files were merged. For this, the parameters "CombineVariants" and "genotypeMergeOptions UNIQUIFY" of the GATK platform were used. The running command was:

**java -jar GenomeAnalysisTK.jar -R human_g1k_v37_decoy.fasta -T CombineVariants -genotypeMergeOptions UNIQUIFY --variant alta_cobertura_limpio.recode.vcf --variant 1000g_karmin_0.1miss.recode.vcf -o todas.vcf**

The resulting file has a total of 7.660.687 variant positions.

4.2 Filtering monomorphic alleles

Since monomorphic alleles are phylogenetically uninformative, we continued to filter those positions from the file. Since the frequency of monomorphic alleles varies with the absence or presence of different individuals, this filter must be applied for all samples together. For this, VCFtools was used and the command run was:

**vcftools --vcf todas.vcf --non-ref-ac-any 1 --out todas_casi_listas --recode**

The file obtained has 10.095 variant positions.

4.3 Depth filters

In this last step in the application of variant filters, we ensured that all variant positions presented a minimum reading depth of two, following the recommendations of [19, 23, 28]. For this we used VCFtools and we ran the following command:

**vcftools --vcf todas_casi_listas.recode.vcf --out todas_listas --min-meanDP 2 --recode**

The resulting file presents a total of 8.839 variant sites.

**Table F.** Summary of data processing applied to file with all sequences and the number of variants conserved per step.

|  | **Number of variant sites** |
| --- | --- |
| Monomorphic allele filter (VCFtools non-ref-ac-any 1) | 10.095 |
| Depth filters (vcftools --min-meanDP 2) | 8.839 |

5. Experimental conditions used for the validation of the new SNPs found

The 13 new sequences of the samples detailed in Table A were used for Sanger validation.

For the quantification of DNA, the concentrations of DNA in solution were obtained from the determination of the absorbance at 260 nm by spectrophotometry. The full spectrum UV visible spectrophotometer was used to quantify and evaluate the purity of DNA, RNA, proteins (NanoDrop 2000) (Thermo Scientific ™). Mili-Q water was used as the sample blank. Subsequently, the necessary dilutions were made to obtain a concentration of 10 ng/µl for each sample.

The amplification products were obtained by PCR amplification using the Eppendorf Mastercycler Nexus thermal cycler (Eppendorf, Germany) and the Biometra T3000 thermal cycler (Biometra, Germany).

The primers were designed with the Primer3 [29] and Oligoanalyzer (IDT) [30].

The PCR assays were optimized by varying the annealing temperature of the primers or by changing polymerase concentration, with a final reaction volume of 25 µl.

The concentrations of the reagents used in the Reaction Mix are detailed below:

**Table H.** **PCR conditions used for Taq GO.**

| **Reagent** | **1X (µl)** | **3.5X** |
| --- | --- | --- |
| Buffer GO 5X | 5 |  |
| dNTPs 2mM | 0.4 | 0.03 mM |
| Primer Fw 2.5 µM | 0.25 | 0.25 µM |
| Primer Rv 2.5 µM | 0.25 | 0.25 µM |
| Taq GO | 0.125 |  |
| DNA | 1 | 10ng/µl |
| H_2_O | 17.975 |  |
| Final volume | 25 |  |

**Table I. PCR conditions used for Taq platinum.**

| **Reagent** | **1X (µl)** | **3.5X** |
| --- | --- | --- |
| Buffer 10X | 2.5 | 8.75 |
| MgCl2 (50mM) | 1 | 2 mM |
| dNTPs 2mM | 0.4 | 0.03 mM |
| Primer Fw 2.5 uM | 0.25 | 0.25 µM |
| Primer Rv 2.5 uM | 0.25 | 0.25 µM |
| Taq Platinum | 0.125 |  |
| DNA | 1 | 10ng/µl |
| H_2_O | 19.5 |  |
| Final volume | 25 |  |

**Figure A. Scheme of the PCR program used.**

The same program was used for both polymerases, where only the annealing temperatures (X ° C) were varied.

The DNA fragments obtained were verified by 1.5% agarose gel electrophoresis (Genbiotech). The gels were prepared by melting an agarose solution in 1X TBE buffer (Tris Base, boric acid, EDTA, pH 8). Before being seeded on the agarose gel DNA samples were diluted in 6X seeding buffer containing GelRed. To estimate the size of the DNA fragment for electrophoresis, the 100 bp marker (Genbiotech) was used. Electrophoresis conditions were modified depending on the size of the nucleic acid fragment to be resolved. In this sense, in all cases a 1X TBE buffer solution was used as a running buffer, while the voltage of the different electrophoreses was constant at 90 Volts. The separation time was also variable between 45-60 minutes. The subsequent visualization of the nucleic acids resolved in the agarose gel was carried out in a Gel Doc XR transilluminator (Bio-Rad), taking advantage of the properties of the GelRed which emits fluorescence when irradiated with UV light after being intercalated between the bases of nucleic acid.

The PCR products that after purification with PE (polyethylene glycol) showed clear bands, were sent to Macrogen, Inc. (Seoul, South Korea), following specifications.

The results obtained were analyzed using various tools available online, Chromas software sequence viewer [31], the SNV analyzer and Indel software Indigo [32]. The BLAST software [33] was used to perform alignments.

**6. Historical precedent of sub-lineages belonging to Q-M848**

**Sub-lineage Q-MPB118**

The Aranã population inhabits mostly the Jequitinhonha mesoregion located North of Minas Gerais [34]. During the 16th century, the strong colonization process that took place in Brazil had a great impact on native people, generating several problems such as loss of ethnic identity and decline of various communities [34]. According to the report from the Eloy Ferreira da Silva Documentation Center (CEDEFES), official historiography indicates that the Aranãs became extinct in the 19th century [35]. The ancient Aranãs belonged to the subgroup of Botocudos, part of the Macro-Jê linguistic trunk [36], which were dispersed in the region of Vale do Rio Doce in Minas Gerais [37]. At the end of the 1990s, the current Aranã group inserted into the indigenous movement in search for their identity [37]. Following the advice of institutions and researchers who resorted to historiography, they contributed to the legitimization of the use of the ethnonym Aranã [34]. Contemporary Aranã ethnic recognition by the Brazilian government dates from 2003 [37].

The Xavante people call themselves A'we, and together with the Xerente (self-named Akwe) form an ethnolinguistic group known in anthropological literature as Acuen, belonging to the Jê linguistic family, from the Macro-Jê trunk [38]. The Acuen group lived dispersed in the region today known as Central-West Brazil before the arrival of the Colonists. The name "Xavante" was assigned by the non-Indians in order to differentiate them from the other Acuens, particularly the Xerente [38]. At the beginning of the 18th century, after the discovery of gold in the Central-West region of Brazil, the arrival of miners, conquerors, settlers, and missionaries put pressure on the local indigenous populations causing conflicts, wars, and migrations. Currently the Xavante inhabit various geographically discontinuous territories located in the State of Mato Grosso, some of which are today designated as indigenous territories [39, 40].

**Sub-lineage Q-MPB139**

The Pasto are an indigenous ethnic group that lived with the Quillacingas in the Northern Andean Area, which is currently the department of Nariño, Southern Colombia, and in the province of Carchi, in Northern Ecuador [41]. It is known that the Pasto were under the rule of the Inca Empire shortly before the Spanish conquest in the 16th century [41]. At the time that the Spanish reached what is now the department of Nariño, this territory was inhabited by a large number of different ethnic groups. The Andean region of Nariño presents archaeological gaps regarding the definition of pre-Hispanic cultural processes and currently the Pasto are an ethnic group in search of archaeological identity [42].

In pre-Columbian times, the Uro ethnic group from Puno, Peru, was distributed along extensive territories of the Andean Altiplano or plateau of the Titicaca Lake, which includes territories of Bolivia, Peru and neighboring areas of Chile [43] and the inter-Andean valleys of the Pacific basin [44]. The language of the Uro ethnic group in pre-conquest times is known as Uruquilla. This language gradually disappeared after the conquest by the Inca Empire [45], which occurred between the 13th and 16th centuries [46], and then by the Spanish colonization in the 16th century, when Quechua and Aymara languages were imposed to facilitate administrative and evangelization activities [47, 48]. Currently, the Uruquilla language is considered extinct [49], and most of the residents of the Altiplano speak Aymara, Quechua (both considered as "sister" languages ​​of the Andean family), and Spanish. The Uro population has drastically decreased as a result of the Inca and Spanish rules [50]. Currently they are distributed in four different settlements dispersed throughout the aquatic areas of the Altiplano, and are known in Bolivia as Uru-Chipaya, Uru-Poopo and Uru-Irohito, and in Peru as the Uros from Puno [51, 52]. The Uro area of Peru is made up of floating islands in the Puno Bay of Titicaca Lake [53].

**Sub-lineage Q-B46**

In the middle of the 16th century, the Spanish colonizers named all the autochthonous peoples that existed in Northwestern Argentina as Colla upon their arrival. The peoples of Northwestern Argentina progressively suffered an acculturation that began with the Inca conquest and deepened with the Spanish colonizers, so their culture was displaced and many simply identify themselves as Collas, ignoring their specific origin. It is known that the members of the Colla people are heirs of the first settlers who inhabited the Andean region, and later they were part of the Tawantinsuyu, the great Inca state that reached its heyday in the 15th century. The Southeast of the Inca Empire was called Collasuyu, a term that comes from the name of a large ethnic group from Titicaca Lake, where people were mostly but not exclusively Aymara. Part of the Andean region corresponding to the current Northwest Argentina included a variety of ethnic groups: Chichas, Atacamas, Casabindos, Cochinocas, Lipez, Atapamas and Uros in the Puna; Omaguacas, Uquías, Tilcaras or Fiscaras or Tiscaras, Purmamarcas and Tilianes in the Quebrada de Humahuaca; Ocloyas, Paypayas, Churumatas, Gaypetes, Osas, Yalas, Azamatas, Tomatas, Omanatas and Yapanatas in the Andean foothills [54].

**Sub-lineage Q-Z35505 / Q-Z35497 / Q-B43**

The Wichi community is one of the most numerous native populations inhabiting the Argentine territory. Traditionally, they inhabit the Chaco (monte chaqueño) and their main activities are fruit gathering, hunting, fishing, and some agricultural practice [55]. The Wichi language is one of the four members of the Mataco-Mataguayan linguistic family [56]. In an attempt to calculate the temporal depth of the Mataco-Mataguayan proto-language, glottochronological calculations were carried out, yielding a minimum date of 17 centuries of internal divergence [57]. Before the arrival of Europeans in the 16th century, this linguistic family encompassed extensions within the Great Central and Southern Chaco, particularly regions that include the Southern part of Bolivia at the height of the Pilcomayo River, the Argentine Northeast including Northeast Salta and Jujuy, Northwestern part of Chaco following the course of the Bermejo River bordering Formosa, Northwestern Formosa and Northwestern Paraguay including the triple border. At the time of the first contact with Europeans, the Mataguayan peoples seem to have lived within the aforementioned limits. The current Mataguayo-Maká group stayed outside these limits and moved near the city of Asunción after the Chaco war (1932-1935) [56].

Another ethnic group belonging to this sub-lineage is the Paresí from Mato Grosso, Brazil. Paresí native people call themselves Halití (people from town) and speak a language that is classified by linguists as belonging to the Arawak family [58]. The Paresí language is related to the Maipure branch of the Arawak family, and a chronological depth of about 3,000 years is estimated [59]. The Paresí are divided into 4 different subgroups: Kazíiniti, Waimaré, Warére and Káwali, and inhabited territories with well-defined limits within an extensive plateau that runs from the headwaters of the Arinos and Paraguay rivers to the headwaters of the Guaporé and Juruena rivers, in the Midwest of the state of Mato Grosso. Written records of the presence of Paresí at the site date back to the 18th century [60, 61]. Until the beginning of the European colonization, the Paresí occupied this extensive territory; currently they are contained within politically delimited areas as indigenous lands, with different and reduced size, and environmental diversity. Even today, they occupy parts of their traditional territory, encompassing the location of its myth of origin: Ponte de Pedra, located 70 km from the city of Campo Novo dos Paresís. According to Paresí cosmology, "in Ponte da Pedra, the humanity, the world, would have begun"; it represents a sacred region for the Paresí people [62].

The Gran Chaco is located in the center of South America; it is the second largest biome in South America, after the Amazon, and is shared by four countries, Paraguay, Bolivia, Brazil, and Argentina. Currently, the Gran Chaco presents patches of wildlife under increasing stress from demographic expansion and economic exploitation [63]. Its area limits to the North with the Amazon; the transition zone is constituted by the plains of Chiquitos; to the East it limits with the Brazilian plateau and its extensions in the Paraguayan Paraneña and the missionary plateau; the limit with this zone is constituted by the rivers Paraguay and Paraná. It borders the Pampa (Argentina) to the South, and is separated from it by the Salado River and the Mar Chiquita lagoon, and to the West by the Andean and sub-Andean region. Little is known about the origin of the different ethnic groups of the Gran Chaco and archaeological data are scarce due to the geography of this region characterized by savannas and meadows flooded by rivers, with fire cycles [64, 65]. The region is believed to have been inhabited for the last 4,000 to 5,000 years, and had previously been a huge swamp [66]. Chaco archaeology has been referenced in close connection with the Andean and/or Amazon Area [67]. Gran Chaco inhabitants have been nomad hunter-gatherers [68] and practiced animal and plant exploitation in microenvironments watered by rivers [69], and some horticultural activities [65]. These populations have been grouped into various ethnolinguistic groups, named Mataguayos, Guaycurú, Tupí-Guaraní, Maskoy, Zamuco, and Lule-Vilela [70]. Throughout the centuries, this region served as a congregation point and refuge for local populations when Europeans arrived in the Americas, as well as for others who settled there as a result of colonial pressure. It was only at the beginning of the 20th century that countries claiming to have a right to the region gained territorial control and their massive presence forced natives into sedentarism [71].

**Sub-lineage Q-CTS11357 / Q-M925**

The Pima identify themselves as Akimel O'odham, which means "people of the river". The name Pima is derived from the native phrase pi-nyi-match, which means "I don't know", applied by Akimel O'odham in response to questions from the Spanish colonizers. The dialect used by this ethnic group is known to belong to the Uto-Aztecan linguistic family. The Akimel O'odham occupied ancestral lands now mapped as part of United States’ Southern Arizona and Mexico’s Northern Sonora. They were divided into two main groups historically called Pima Alto and Pima Bajo. The Alto Pima lived along the Gila and Salt rivers. The Pima Bajo lived along the Yaqui and Sonora rivers much further South [72].

The Aztecs are also named Nahua. Like the Pima, the Nahua also belong to the Uto-Aztecan linguistic family. Today this family of languages ​​exhibits an exceptionally large North-South geographic range, from Southern Idaho, United States, to El Salvador and Nicaragua in Central America. The Uto-Aztecan linguistic family is also notable due to the variety of cultural adaptations among speaking communities, such as the Shoshone, gatherers in the United States, to the builders of urban states such as the Nahua in Mesoamerica [73]. The standard account of the origins of this language family reports that the Uto-Aztecan began as foragers in Southwest United States and Northwest Mexico [74-76].

The Karitiana are a Native American group still little studied by anthropologists. Their Indigenous Territory is located today in the municipality of Porto Velho, Rondônia state, North Brazil. The origin or etymology of the word Karitiana is not known; indigenous people themselves state that it was attributed to them by rubber tappers who entered their territory in late 19th and early 20th centuries. They call themselves Yjxa (translated as we or people). Their first contacts with European-descendent people probably occurred at the end of the 18th century, and later intensified with the mass arrival of the rubber tappers by the end of the 19th century [77]. The Karitiana language belongs to the only remnant of the Arikém linguistic family, this being a subfamily of the Tupí linguistic trunk. The Tupí is considered one of the linguistic groups occupying the largest geographic extension in South America, dispersing both in the Amazon and in La Plata Basin. According to glottochronological dating, Proto-Tupí, the root language of the components of the Tupian line originated about 5,000 years ago [78, 79]. Furthermore, given the concentration of Tupí linguistic branches, it has been suggested that the state of Rondônia, in the Amazon region of Brazil, is probably the place of origin for Proto-Tupí [80].

**Sub-lineage Q-Y27993/Q-Y27992**

Chané origin is unknown and controversial since Chané themselves claim to be autochthonous from that region but, on the other hand, history researchers suppose that the Chané arrived in Itiyuro escaping from the domain of Guaraní-speaking groups [81]. The language spoken by the Chané belongs to the Arawak linguistic stock, geographically this language is distributed from the Antilles and Bahamas to the lowlands of South America in Argentina and from the mouth of the Amazon River to the foothills of the Andes [82]. Linguistic studies carried out to date proto-languages estimated 4,461-4,085 BP for the proto-Arawak language [83]. It has been suggested that the Northwest of the Amazon is the Arawak homeland based on the lexical diversity of this linguistic family in that geographic center; yet it has also been proposed that it could be the Atlantic coast or the Western Amazon [84]. The Arawak are known as hunter-gatherers and peaceful farmers; their most important crops were cassava, corn, potatoes, sweet potatoes, beans, peanuts, peppers, cotton, and tobacco. They were good canoeists and had boats that could hold up to 100 people; these boats were also used for trading with Caribbean peoples, and different communities in South and Central America. The Arawak also traded with the North American people of the Florida coast, such as Timucua and Calusa [72]. As reported by other authors, the Chané may have reached the Argentine territory in more recent times, probably escaping from the Guaraní about 2,000 years before the Common Era (BCE) [81] since, according to archaeological data, the Guaraní occupation process may have begun in 2,000 BCE, and did not respect the populations of the conquered regions [85]. The Guaraní were essentially Amazonian cultures, so Arawak groups may have arrived in Salta through migrations from Bolivia, fleeing from the Chiriguanos (Guaraní branch) who occupied the Bolivian territory [81], or from Eastern Paraguay where archaeological sites show regional sequences evidencing the continuous and dense Guaraní presence [85].

**Sub-lineage Q-Z19357**

It is known that previously to the European conquest the various Maxakalí groups occupied an area between the Pardo and Doce rivers, corresponding to Southeastern Bahia, Northeastern Minas Gerais, and Northern Espírito Santo, in Brazil. The remnants of these groups, currently known as Maxakalí, live today in two indigenous areas: Água Boa and Pradinho, unified in the Maxakalí Indigenous Land, at the head of the Umburanas River, in Northeastern Minas Gerais. Currently, Maxakalí groups use their native language, belonging to the Macro-Jê linguistic trunk. Since the occupation of the Umburanas region by cattle ranchers, there has been a decline in Maxakalí population due to the deterioration in their quality of life and conflicts with the farmers of the region [86].

The Macro-Jê language family has been proposed as a language group that includes the Jê family and several others, including the Maxakalí [87-89]. Although many Macro-Jê languages ​​are spoken in the Brazilian Amazon, the geographical distribution is rather circum- Amazonian, encompassing the Brazilian Cerrado ecoregion, with borders in the Amazon region to the North and the Gran Chaco to the West and Southwest. Most of the Macro-Jê languages ​​are concentrated in Eastern and Northeastern Brazil, although a few groups inhabit the Center and Southwest of Brazil. The only Macro-Jê language known to be spoken outside of present-day Brazil is Otúke, which was spoken East of the Paraguay River in Bolivia [89].

**Sub-lineage Q-MPB016**

A native group of people who call themselves Hupda inhabit scattered villages in the upper basin of the Negro River, Northwest Amazonia, living on hunting, fishing, gathering, and small-scale cassava cultivation for subsistence. The Hup language (also known as Hupda or Jupde) is spoken in the Vaupés region of Northwestern Amazonia, an area of ​​approximately 2,000 square kilometers that straddles the border between Brazil and Colombia [82]. The Hup language belongs to the small Makú linguistic family. As far as it is known, this family has no relation with the Tukano or Arawak language families, who also inhabit the Northwestern Amazonia, except for some obvious and few linguistic loans [90, 91]. Little is known about the history of the Makú in pre-conquest times. It is believed that human occupation in the Northwestern Amazonian area probably occurred first, with Makú settled in the interfluvial areas, in mainland "patches", followed by the Arawak and the Tukano, who settled in the high ravines of the rivers [91].

Given the poor lexical information, the classification of the Makú linguistic family presents differences according to the source [88, 92-95]. Furthermore, the family itself has at least four alternative names, in addition to Makú or Macú [88, 93], it has been named Puinavean [95, 96], Vaupés-Japura (Uaupés–Japura) [97] and Nadahup [92]. On the other hand, since the term Makú, of Arawak origin, has a pejorative connotation, it is rejected by its speakers. There is no common self-denomination adopted by the Makú group itself [98]. Due to the influence of the indigenous movement in the Río Negro region since the mid-80’, pejorative names such as Makú have fallen into disuse [91, 99].

The term Cañari appears in the first Spanish chronicles in reference to the inhabitants who occupied a large area within the current territory of Ecuador. Although there are several hypotheses about the meaning of the word "cañari" and the time when this term appeared, little is known about its history and the language that this ethnic group spoke. Archaeology has preferred the term “proto cañari” [100] and has traditionally classified the proto cañari chronology into two successive phases: Tacalshapa (300 -1,200 before our Era) and Cashaloma (1,000 to 1,500 of our Era), which were defined after the finding of two different types of ceramics in archaeological sites of the Ecuadorian Austro [101]. According to the Spanish chronicles, although they shared a common language and traditions, the Cañaris would have divided into several political nuclei, each one managed by its own leader and settled in its own valley. These nuclei would also have been in permanent contact with each other through products exchange, but also conflicts [102].

The Incas carried out a massive conquest in the Andes, and reached the Cañari territory under the command of the sovereign Túpac Yupanqui around 1463 [103-105]. In order to avoid any attempt to rise, and to improve labor conditions, this area was subject to population displacements to and from the Cañari territory. These displaced people are known under the name of mitmakuna or mitimaes [103, 106]. Nevertheless, the Incas only managed with difficulty to take control of the Cañari territory, a task that was also interrupted by the arrival of the Spanish in this area in 1533 [101, 104]. Regarding their development, some authors suggest that the Inca and Spanish wars of conquest could have destroyed the pre-Columbian “Cañaris”, which ould explain the almost total “Incaization” and “Hispanicization” of those who currently claim to be Cañaris [105]. Other authors, while recognizing incaization and hispanicization, propose rather that the Cañaris did not disappear, but voluntarily chose to adopt Inca and Spanish customs in order to survive culturally [107].

References

1. Jurado Medina LS. Tipificación de marcadores uniparentales en poblaciones mestizas de Argentina: Universidad Nacional de La Plata, Facultad de Ciencias Naturales y Museo. Tesis doctoral. Available from: <http://sedici.unlp.edu.ar/bitstream/handle/10915/49551/Documento_completo.pdf-PDFA.pdf?sequence=5&isAllowed=y>; 2015.

2. Jurado Medina LS, Ramallo V, Calandra H, Lamenza G, Braunstein J, Salceda S, et al. Linajes paternos del Gran Chaco, un abordaje desde el ADN. Folia Histórica del Nordeste. 2014.

3. Jurado Medina LS, Paz Sepulveda PB, Ramallo V, Sala C, Beltramo J, Schwab M, et al. Continental origin for Q Haplogroup Patrilineages in Argentina and Paraguay. Human Biology Open Access Pre-Prints 177 <https://digitalcommonswayneedu/humbiol_preprints/177>. 2020.

4. GATK (Genome Analysis Toolkit). Available from: <https://gatk.broadinstitute.org/hc/en-us>.

5. McKenna A, Hanna M, Banks E, Sivachenko A, Cibulskis K, Kernytsky A, et al. The Genome Analysis Toolkit: a MapReduce framework for analyzing next-generation DNA sequencing data. Genome research. 2010;20(9):1297-303.

6. Andrews S, &-Babraham-Bioinformatics. FastQC: A quality control tool for high throughput sequence data. Manual. 2010. Available from: <http://www.bioinformatics.babraham.ac.uk/projects/fastqc/>.

7. Simon A. FASTQC software Babraham Bioinformatics. Available from: <http://www.bioinformatics.babraham.ac.uk/projects/fastqc/>.

8. Schmieder R, Edwards R. Quality control and preprocessing of metagenomic datasets. Bioinformatics. 2011;27(6):863-4.

9. PRINSEQ software. Available from: <https://sourceforge.net/projects/prinseq/>.

10. Genome Reference Consortium Human Build 37 (GRCh37). Available from: <https://www.ncbi.nlm.nih.gov/genome/guide/human/>.

11. Li H, Durbin R. Fast and accurate short read alignment with Burrows–Wheeler transform. bioinformatics. 2009;25(14):1754-60.

12. Li H, Durbin R. Fast and accurate long-read alignment with Burrows–Wheeler transform. Bioinformatics. 2010;26(5):589-95.

13. Picard. Available from: <https://broadinstitute.github.io/picard/>.

14. Samtools. Available from: <https://samtools.github.io/hts-specs/SAMv1.pdf>.

15. Samtools Manual. Available from: <http://www.htslib.org/doc/1.6/samtools.html>.

16. Consortium GP. A global reference for human genetic variation. Nature. 2015;526(7571):68.

17. DePristo MA, Banks E, Poplin R, Garimella KV, Maguire JR, Hartl C, et al. A framework for variation discovery and genotyping using next-generation DNA sequencing data. Nature genetics. 2011;43(5):491.

18. Van der Auwera GA, Carneiro MO, Hartl C, Poplin R, Del Angel G, Levy‐Moonshine A, et al. From FastQ data to high‐confidence variant calls: the genome analysis toolkit best practices pipeline. Current protocols in bioinformatics. 2013;43(1):11.0. 1-.0. 33.

19. Pinotti T, Bergström A, Geppert M, Bawn M, Ohasi D, Shi W, et al. Y chromosome sequences reveal a short Beringian standstill, rapid expansion, and early population structure of Native American Founders. Current Biology. 2019;29(1):149-57. e3.

20. Mallick S, Li H, Lipson M, Mathieson I, Gymrek M, Racimo F, et al. The Simons genome diversity project: 300 genomes from 142 diverse populations. Nature. 2016;538(7624):201.

21. Norstedt J, Solli A, Dawtry K, Reed K, &-Q-Nordic-Family-Tree-DNA-group. Available from: <http://www.familytreedna.com/groups/qnordic/about>.

22. Weeks NT, Luecke GR. Optimization of SAMtools sorting using OpenMP tasks. Cluster Computing. 2017;20(3):1869-80.

23. Hallast P, Batini C, Zadik D, Maisano Delser P, Wetton JH, Arroyo-Pardo E, et al. The Y-chromosome tree bursts into leaf: 13,000 high-confidence SNPs covering the majority of known clades. Molecular biology and evolution. 2015;32(3):661-73. doi: 10.1093/molbev/msu327. PubMed PMID: 25468874; PubMed Central PMCID: PMC4327154.

24. Poznik GD, Henn BM, Yee M-C, Sliwerska E, Euskirchen GM, Lin AA, et al. Sequencing Y chromosomes resolves discrepancy in time to common ancestor of males versus females. Science. 2013;341(6145):562-5.

25. Poznik GD, Xue Y, Mendez FL, Willems TF, Massaia A, Sayres MAW, et al. Punctuated bursts in human male demography inferred from 1,244 worldwide Y-chromosome sequences. Nature genetics. 2016;48(6):593.

26. VCFtools manual. Available from: <http://vcftools.sourceforge.net/man_latest.html>.

27. Karmin M, Saag L, Vicente M, Sayres MAW, Järve M, Talas UG, et al. A recent bottleneck of Y chromosome diversity coincides with a global change in culture. Genome research. 2015;25(4):459-66.

28. Grugni V, Raveane A, Ongaro L, Battaglia V, Trombetta B, Colombo G, et al. Analysis of the human Y-chromosome haplogroup Q characterizes ancient population movements in Eurasia and the Americas. BMC biology. 2019;17(1):3.

29. Primer3. Available from: <http://bioinfo.ut.ee/primer3-0.4.0/>.

30. IDT. Oligoanalyzer. Available from: <https://www.idtdna.com/pages/tools/oligoanalyzer?utm_source=google&utm_medium=cpc&utm_campaign=ga_oligoanalyzer&utm_content=ad_group_oligo_analyzer&gclid=Cj0KCQjwwuD7BRDBARIsAK_5YhUCUENqH-anzBNEZS_InuWLUvfUGPeDEBbGaf4wAfTzXuCDk_l6LicaAuLvEALw_wcB>.

31. Chromas. Available from: <https://technelysium.com.au/wp/chromas/>.

32. Indigo Is. Available from: <https://www.gear-genomics.com/indigo/>.

33. BLAST. Available from: <https://blast.ncbi.nlm.nih.gov/Blast.cgi?PROGRAM=blastn&PAGE_TYPE=BlastSearch&LINK_LOC=blasthome>.

34. Carvalho LC. Os Arana e sua indianidade: disputas internas por legitimidade e o reconhecimento oficial como grupo indígena. tesis, Belo Horizonte 2008.

35. BAETA E. Aranã: a luta de um povo no Vale do Jequitinhonha. Contagem: Cedefes. 2003.

36. Seki L. Apontamentos para a bibliografia da língua Botocudo/Borum. Cadernos de Estudos Lingüísticos. 1990;18:115-42.

37. Cardoso LSdM, Queiroz ACL, Pena JL, Machado-Coelho GLL, Heller L. Aranãs do médio Jequitinhonha: aspectos socioeconômicos, demográficos e sanitários de uma população indígena desaldeada. Ciência & Saúde Coletiva. 2016;21:3859-70.

38. Neel JV, Salzano FM, Junqueira PC, Keiter F, Maybury-Lewis D. Studies on the Xavante indians of the Brazilian Mato Grosso. American journal of human genetics. 1964;16(1):52.

39. Ricardo B, Ricardo F. Povos indígenas no Brasil: 2001/2005: Instituto Socioambiental; 2006.

40. Graham L. Povos Indigenas no Brasil 2008. Equipo de Edición de la Enciclopédia Pueblos Inginenas en Brasil:[Available from: <https://pib.socioambiental.org/es/Povo:Xavante>.

41. Moncayo HS. Origen de los pueblos Pastos 2009. Available from: <http://www.rupestreweb.info/pastos.html>.

42. ARROYO FC. PASTOS Y QUILLACINGAS: DOS GRUPOS ETNICOS EN BUSCA DE IDENTIDAD ARQUEOLOGICA. Revista Colombiana de Antropología. 1992;29.

43. Sandoval JR, Lacerda DR, Jota MS, Salazar-Granara A, Vieira PPR, Acosta O, et al. The genetic history of indigenous populations of the Peruvian and Bolivian Altiplano: the legacy of the Uros. PLoS One. 2013;8(9).

44. Chero L. Potencialidades de una integración real en la cuenca sudamericana del pacífico: Los intentos en Ecuador, Perú, y Chile; y sus retos a comienzos del siglo XX. CA Bello, Integración Regional. 2008;4.

45. Tamayo Herrera J. Nuevo compendio de historia del Perú. Editorial Osiris, Lima, Peru. 1987;372.

46. Sáenz V. VISIONES SOBRE GENTE URU EN BOLIVIA. Revista Textos Antropológicos. 2003;14:55.

47. Canahuire Ccama JA. Evolución histórica y social de las naciones Collas. Editor LACG. 1999:84.

48. Cerrón Palomino R. Examen de la teoría aimarista de Uhle. Max Uhle y el Perú antiguo. 1998.

49. Hannß K. Uchumataqu: The Lost Language of the Urus of Bolivia; a Grammatical Description of the Language as Documented Between 1894 and 1952: CNWS publications; 2008.

50. Censo Peruano 2021. Available from: <https://www.inei.gob.pe/>.

51. Torero A. Acerca de la familia lingüística uruquilla (Uru-Chipaya). Revista Andina. 1992;10(1):171-89.

52. Wachtel N. Men of the water: The Uru problem (XVI and XVII centuries). Cambridge University Press & Editions de la Maison des Sciences de L’Homme; 1986.

53. R CP. Reconstrucción del proto-Uro:fonología. Lexis, v. XXXI/1–2, Lima, 47–104. 2007.

54. KARASIK G, MACHACA R. Kollas de Jujuy. Un pueblo, muchos pueblos Pueblos indígenas en la Argentina cap. 2016;6.

55. Juárez M. Los Wichís Matacos, una cultura aborigen del Gran Chaco argentino: fotografías en blanco y negro de una cultura condenada. 2006.

56. Fabre A. Los pueblos del Gran Chaco y sus lenguas, segunda parte: Los mataguayo. Suplemento Antropológico. 2005;(40):313-435.

57. Swadesh M. Mapas de clasificación lingüística de México y las Américas: Universidad Nacional Atónoma de México; 1959.

58. Montserrat RMF. Línguas indígenas no Brasil contemporâneo. Índios no Brasil Brasília: MEC. 1994:93-104.

59. Urban G. A história da cultura brasileira segundo as línguas nativas. História dos índios no Brasil. 1992;2:87-102.

60. Badariotti N. Exploração no norte de Matto Grosso, região do alto Paraguay e planalto dos Parecis: apontamentos de historia natural, etnographia, geographia e impressões: Escola typ. salesiana; 1898.

61. Silva JVd. A Capitania de Mato Grosso: política de povoamento e população–século XVIII. São Paulo: Tese de Doutorado: DH/FFLCH/USP. 1994.

62. Moi FP, Morales WF. Arqueologia e gestão de recursos culturais entre os Paresi da Chapada dos Pareci, MT (Brasil). Especiaria: Cadernos de Ciências Humanas. 2015;11(20, 21).

63. Krebs E, Braunstein J. The renewal of Gran Chaco studies. History of Anthropology Newsletter. 2011;28(1):9-19.

64. Alvarsson J-A. The mataco of the Gran Chaco. Uppsala: University of Uppsala. 1988.

65. Braunstein J, Miller E. Ethnohistorical introduction. Peoples of the Gran Chaco. 1999;15:22.

66. Demarchi DA, Ministro AG. Genetic structure of native populations from the Gran Chaco region, South America. International Journal of Human Genetics. 2008;8(1-2):131-41.

67. Braunstein JA, Salceda S, Calandra HA, Méndez M, Ferrarini S. Historia de los chaqueños–Buscando en la “papelera de reciclaje” de la antropología sudamericana”. Acta Americana Journal of the Swedish Americanist Society. 2002;10(1):63-93.

68. Saeger JS. The Chaco Mission Frontier: The Guaycuruan Experience: University of Arizona Press; 2000.

69. Mendoza M. Range area and seasonal campsites of Toba bands in western Chaco, Argentina. Before Farming. 2003;2003(4):1-12.

70. Loukotka Č, Wilbert J. Classification of South American indian languages: Latin American Center, University of California, Los Angeles; 1968.

71. Braunstein J, Vidal A. En prensa. The Gran Chaco: convergence of languages and peoples. The Languages of Hunter-Gatherers. Historical and global perspectives ….

72. Waldman C. Encyclopedia of native American tribes: Infobase Publishing; 2006.

73. Hill JH. Proto‐Uto‐Aztecan: a community of cultivators in Central Mexico? American Anthropologist. 2001;103(4):913-34.

74. Campbell L. American Indian languages: the historical linguistics of Native America: Oxford University Press on Demand; 2000.

75. Fowler CS. Some lexical clues to Uto-Aztecan prehistory. International Journal of American Linguistics. 1983;49(3):224-57.

76. Hale K, Harris D. Historical linguistics and archaeology. Handbook of North American Indians. 1979;9:170-7.

77. Luciana S, Ferreira VVF. Karitiana, Povos Indigenas no Brasil 2018. Available from: <https://pib.socioambiental.org/es/Povo:Karitiana>.

78. Rodrigues AD. Classification of Tupi-Guarani. International Journal of American Linguistics. 1958;24(3):231-4.

79. Rodrigues ADI, Hauptmann A, Hartmann T. A classificação do tronco lingüístico Tupi. Revista de Antropologia. 1964:99-104.

80. Dixon RM, Aikhenvald AY. The amazonian languages: Cambridge University Press Cambridge; 1999.

81. Combès I. De Sanandita al Itiyuro: los chanés, los chiriguanos (¿ y los tapietes?) al sur del Pilcomayo. Indiana. 2007;24:259-89.

82. Epps P. Language classification, language contact, and Amazonian prehistory. Language and Linguistics Compass. 2009;3(2):581-606.

83. Brown CH, Clement CR, Epps P, Luedeling E, Wichmann S. The Paleobiolinguistics of Domesticated Manioc (Manihot esculenta). Ethnobiology Letters. Vol. 4 (2013):pp. 61-70 (10 pages).

84. Walker RS, Ribeiro LA. Bayesian phylogeography of the Arawak expansion in lowland South America. Proceedings of the Royal Society B: Biological Sciences. 2011;278(1718):2562-7.

85. Noelli FS. La distribución geográfica de las evidencias arqueológicas guaraní. Revista de Indias. 2004;64(230):17-34.

86. Baqueiro-Paraiso. Maxakalí, Povos indígenas em Minas Gerais 2018. Available from: <https://pib.socioambiental.org/pt/Povo:Maxakali>.

87. Mason J. Alden: The languages of South American Indians. Handbook of South American Indians, Bd. 6. Bull Bur Amer Ethnol. 1950;(143).

88. Rodrigues ADI. Línguas brasileiras: para o conhecimento das línguas indígenas: Edições Loyola; 1994.

89. Rodrigues AD. Macro-jê. The amazonian languages. 1999:165-206.

90. Martins V. Reconstrução fonológica do Protomaku oriental: Netherlands Graduate School of Linguistics; 2005.

91. Pozzobon J. maku (1999). Instituto Socioambiental-ISA Recuperado el. 26(10):07.

92. Epps P. Grammatical borrowing in Hup. Grammatical Borrowing: A Cross-linguistic Survey. Berlin: Mouton de Gruyter.2008 b.

93. Martins SA, Martins V. Makú. The Amazonian Languages, ed. RMW Dixon and Alexandra Y. Aikhenvald. Cambridge: Cambridge University Press; 1999.

94. Kaufman T. The native languages of South America. Atlas of the World's Languages, ed. Christopher Mosely and RE Asher. New York: Routledge; 1994.

95. Campbell L. American Indian Languages. The Historical Linguistics of Native America. 198 Madison Avenue, New York, New York 10016: First published in 1997 by Oxford University Press, Inc.; 1997.

96. Kaufman T. Language history in South America: What we know and how to know more. Amazonian linguistics: Studies in lowland South American languages. 1990:13-67.

97. Ramirez H, editor Família Makú ou família Uaupés-Japura. meeting of ANPOLL, Belém, Brazil; 2001.

98. Becerra GC. Viviendo en el bosque. Un siglo de investigaciones sobre los makú del Noroeste amazónico: Universidad Nacional de Colombia; 2015.

99. Lolli PA. A plasticidade Maku. Ilha Revista de Antropologia. 2016;18(2):177-98.

100. Idrovo J, Gomis D. Historia de una región formada en el Austro del Ecuador y sus conexiones con el norte del Perú. Imprenta América Latina, Cuenca. 2009:41-4.

101. Illescas CAL. TRADICIÓN CERÁMICA Y OCUPACIÓN PRECOLOMBINA DEL PIEDEMONTE ORIENTAL DE LOS ANDES: EL CASO DEL VALLE DEL RÍO CUYES (MORONA SANTIAGO, ECUADOR). Anales de la Universidad Central del Ecuador. 2016;1(374).

102. Gauiria Md. Relación que envió a mandar su majestad se hiciese de esta ciudad de Cuenca y de toda su provincia (Cañaribamba). In Relaciones Geográficas de Indias – Perú. Atlas, pp 281–87. 1965;Madrid: Jiménez de la Espada.

103. Itier C. Les Incas: Les Belles lettres; 2008.

104. Pablos H. Relación que envió a mandar Su Magestad se hiziese desta ciudad de Cuenca y de toda su provincia. Relaciones Geográficas de Indias: Perú. 1965;2:265-70.

105. Hirschkind L. Historia de la población indígena del Cañar. Revista de Antropología. 2013;20:41-78.

106. Cieza de León P. La crónica del Perú [1553]. Ed M Ballesteros. 1962.

107. Salomon F. Ancestros, huaqueros y los posibles antecedentes del “Incaísmo” cañari. Revista de Antropología. 2013:7–40.
