# Supplementary figures and images for: Human Y chromosome sequences from Q Haplogroup reveal a South American settlement pre-18,000 years ago and a profound genomic impact during the Younger Dryas
Source: PLoS One. 2022 Aug 17;17(8):e0271971. doi: 10.1371/journal.pone.0271971 (PMC9385064; doi:10.1371/journal.pone.0271971)

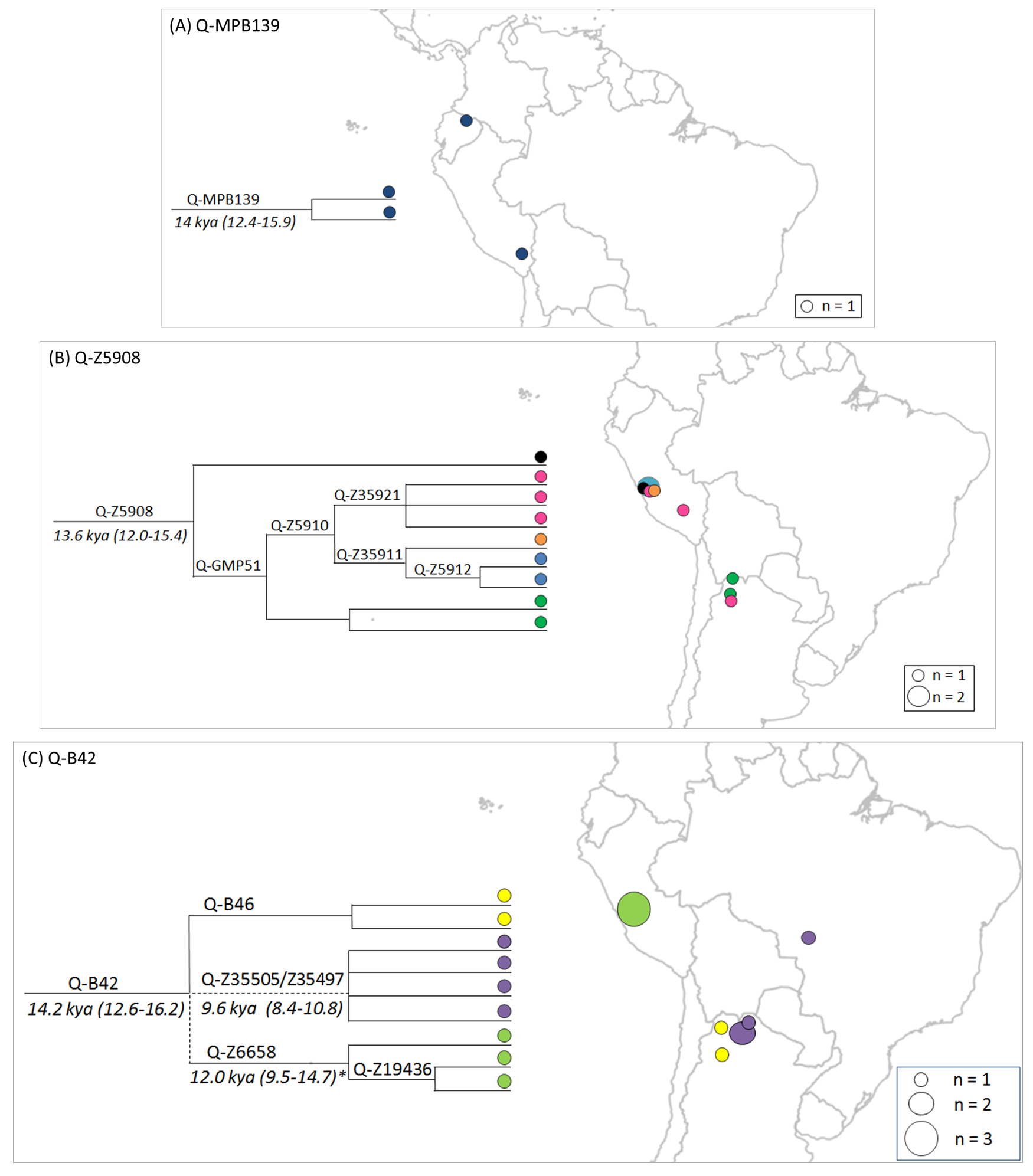

Supplement: S1 Fig — Colored circles represent the geographic distribution and sub-lineage membership, as shown in the inset tree. The size of the circles is related to the number of subjects and is specified with the "n" in the box to the right, see S1 Table. Estimated sub-lineage divergence times are represented in italics, in kya, and with a 95% confidence interval between parentheses (for more details see Methods), those shown without asterisks are dates estimated in this study, those with an asterisk are taken from the literature, see S5 Table. Nodes that do not present dates are those that could not yet be estimated. Dotted lines indicate that their phylogenetic link still needs to be further studied and confirmed. Layer map downloaded from [100]. (PNG) [file pone.0271971.s002.png]

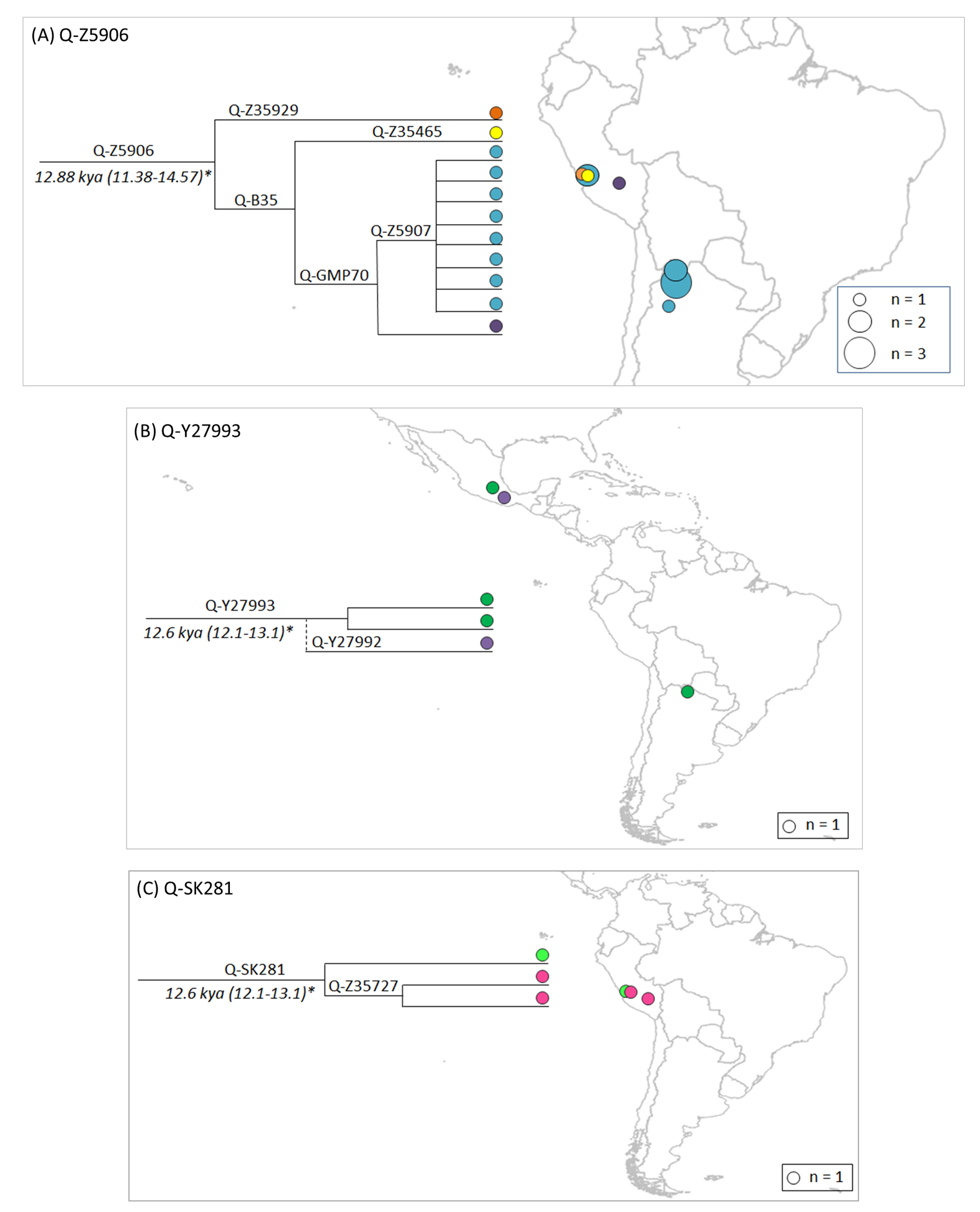

Supplement: S2 Fig — Colored circles represent the geographic distribution and sub-lineage membership, as shown in the inset tree. The size of the circles is related to the number of subjects and is specified with the "n" in the box to the right, see S1 Table. Estimated sub-lineage divergence times are represented in italics, in kya, and with a 95% confidence interval between parentheses (for more details see Methods), those shown without asterisks are dates estimated in this study, those with an asterisk are taken from the literature, see S5 Table. Nodes that do not present dates are those that could not yet be estimated. Dotted lines indicate that their phylogenetic link still needs to be further studied and confirmed. Individuals with Mexican ancestry from Los Angeles have been arbitrarily represented in City of Mexico (S1 Table). Layer map downloaded from [100]. (PNG) [file pone.0271971.s003.png]

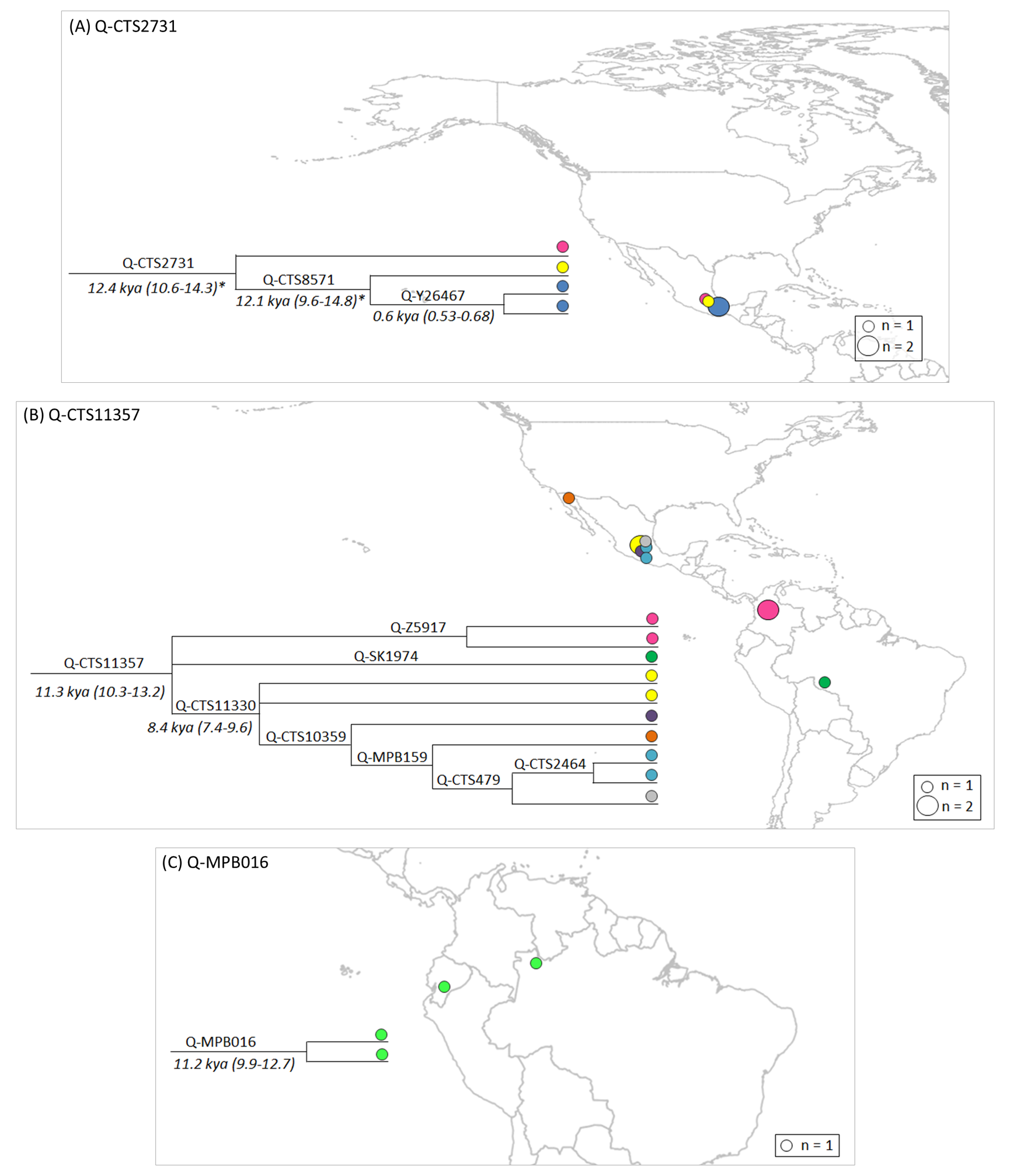

Supplement: S3 Fig — Colored circles represent the geographic distribution and sub-lineage membership, as shown in the inset tree. The size of the circles is related to the number of subjects and is specified with the "n" in the box to the right, see S1 Table. Estimated sub-lineage divergence times are represented in italics, in kya, and with a 95% confidence interval between parentheses (for more details see Methods), those shown without asterisks are dates estimated in this study, those with an asterisk are taken from the literature, see S5 Table. Nodes that do not present dates are those that could not yet be estimated. Dotted lines indicate that their phylogenetic link still needs to be further studied and confirmed. Individuals with Mexican ancestry from Los Angeles have been arbitrarily represented in City of Mexico (S1 Table). Layer map downloaded from [100]. (PNG) [file pone.0271971.s004.png]

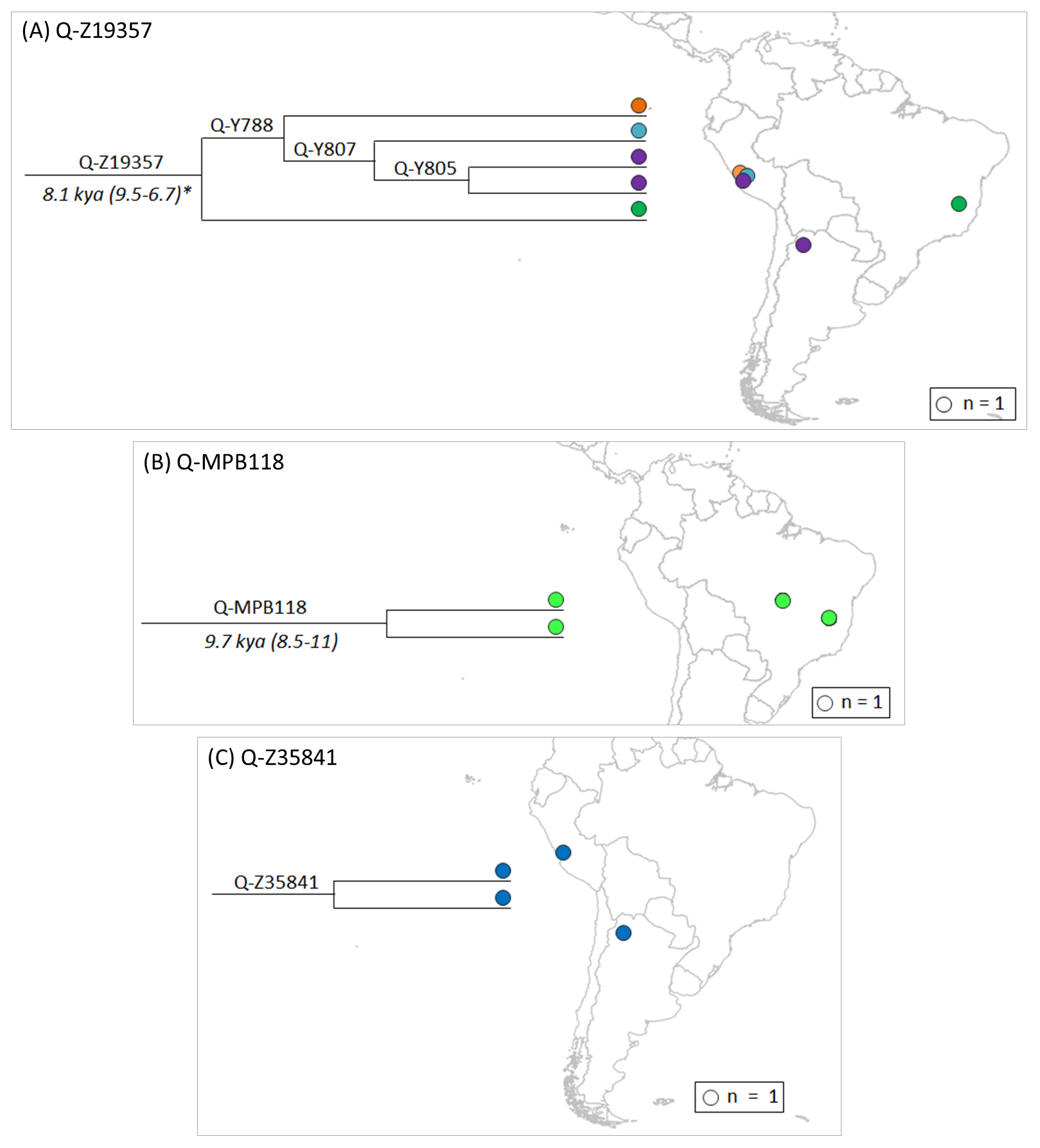

Supplement: S4 Fig — Colored circles represent the geographic distribution and sub-lineage membership, as shown in the inset tree. The size of the circles is related to the number of subjects and is specified with the "n" in the box to the right, see S1 Table. Estimated sub-lineage divergence times are represented in italics, in kya, and with a 95% confidence interval between parentheses (for more details see Methods), those shown without asterisks are dates estimated in this study, those with an asterisk are taken from the literature, see S5 Table. Nodes that do not present dates are those that could not yet be estimated. Dotted lines indicate that their phylogenetic link still needs to be further studied and confirmed. Layer map downloaded from [100]. (PNG) [file pone.0271971.s005.png]

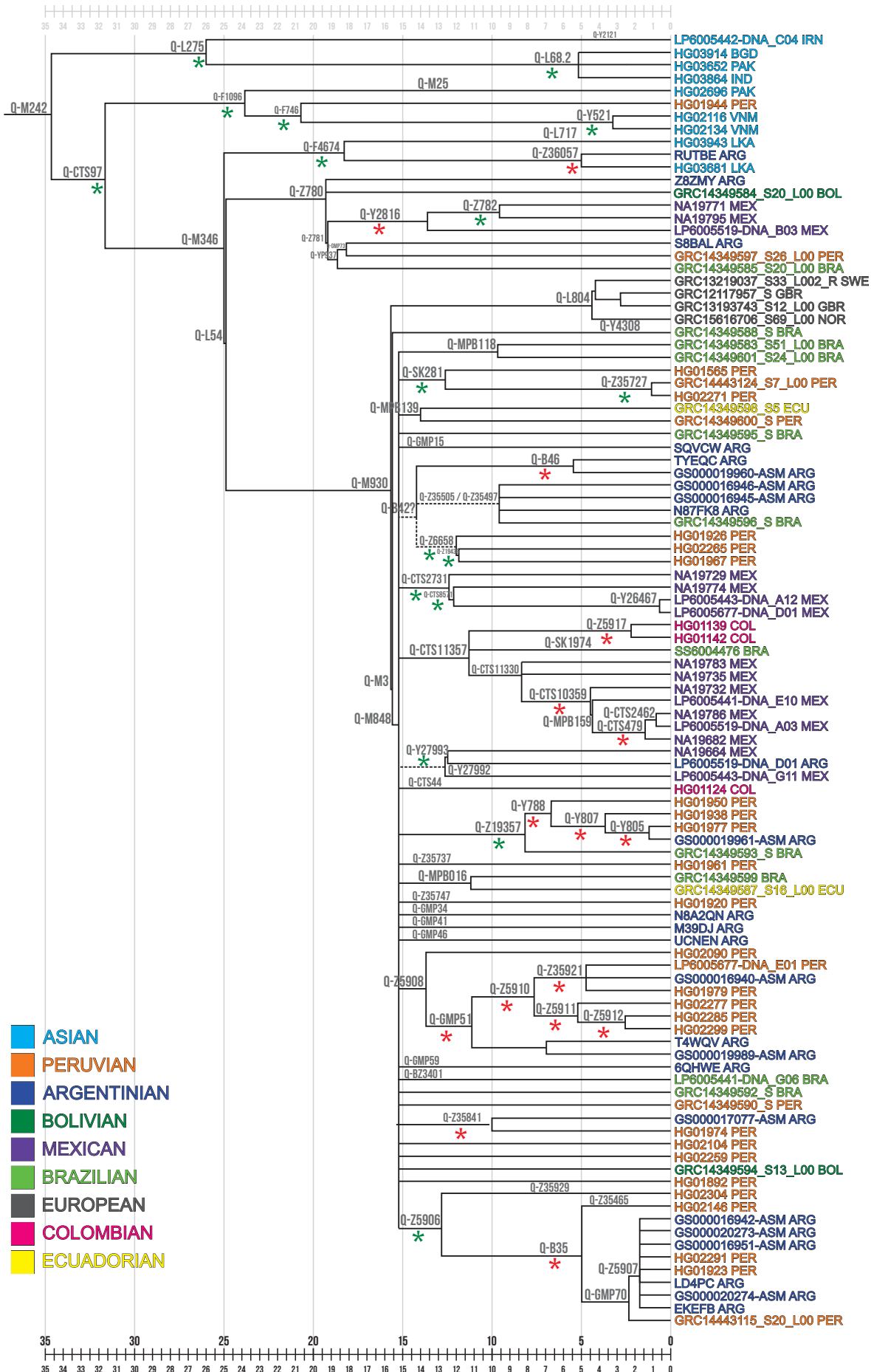

Supplement: S5 Fig — The colors of each individual of the tree are according to the macro-area or the country according to the box on the left. The dashed lines are used to represent branches that require to be further studied for a better definition. The length of the branches is represented as a function of time in kya according to the axis represented. The nodes without asterisks are those dated in this study. The red asterisks are the nodes that could not be dated and the length of their branches does not represent a defined time depth. The green asterisks represent the dates taken from the literature; for a complete list of the dates used in this figure, see S5 Table. For more information about data set, see S1 Table and Table A in S1 Text. (PDF) [file pone.0271971.s006.pdf]
