# Supplementary material for: Human Y chromosome sequences from Q Haplogroup reveal a South American settlement pre-18,000 years ago and a profound genomic impact during the Younger Dryas
Source: PLoS One. 2022 Aug 17;17(8):e0271971. doi: 10.1371/journal.pone.0271971 (PMC9385064; doi:10.1371/journal.pone.0271971)
Supplement: S6 Fig — The length of the branches is expressed as a function of the number of SNPs found in each one. The asterisk indicates samples available only as VCF (see Section 3 in S1 Text and S1 Table). (PDF) [file pone.0271971.s007.pdf]

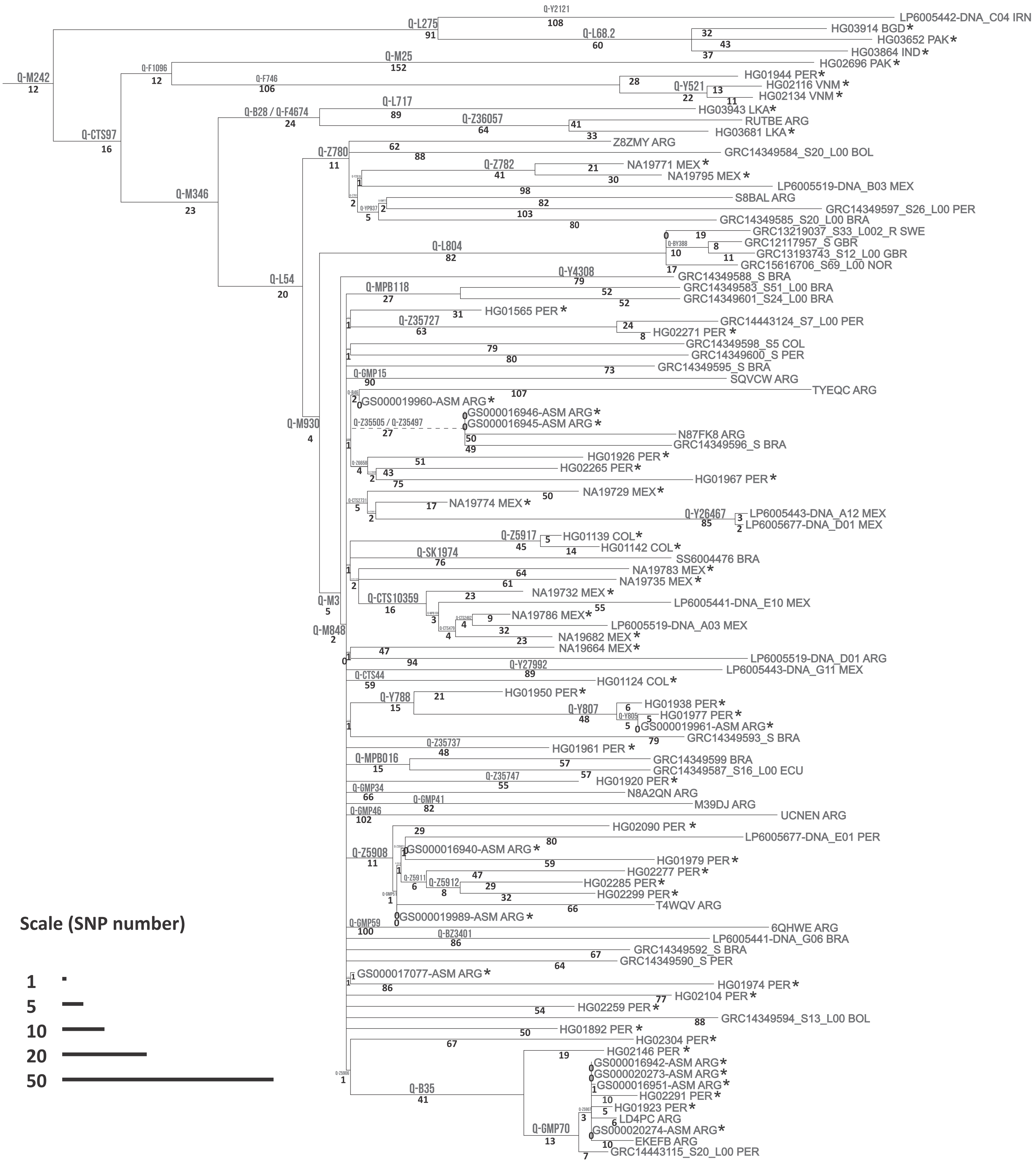

Q-M242
Q-L275
Q-Y2121
Q-L68.2
Q-M25
Q-F1096
Q-F746
Q-Y521
Q-CTS97
Q-B28 / Q-F4674
Q-L717
Q-Z36057
Q-M346
Q-Z780
Q-Z782
Q-L804
Q-L54
Q-Y4308
Q-MPB118
Q-Z35727
Q-GMP15
Q-M930
Q-Z35505 / Q-Z35497
Q-Y26467
Q-Z5917
Q-SK1974
Q-CTS10359
Q-M3
Q-M848
Q-Y27992
Q-CTS44
Q-Y788
Q-Y807
Q-Z35737
Q-MPB016
Q-Z35747
Q-GMP34
Q-GMP41
Q-GMP46
Q-Z5908
Q-Z5912
Q-GMP59
Q-BZ3401
Q-B35
Q-GMP70
LP6005442-DNA_C04 IRN
HG03914 BGD*
HG03652 PAK*
HG03864 IND*
HG02696 PAK*
HG01944 PER*
HG02116 VNM*
HG02134 VNM*
HG03943 LKA*
RUTBE ARG
HG03681 LKA*
Z8ZMY ARG
GRC14349584_S20_L00 BOL
NA19771 MEX*
NA19795 MEX*
LP6005519-DNA_B03 MEX
S8BAL ARG
GRC14349597_S26_L00 PER
GRC14349585_S20_L00 BRA
GRC13219037_S33_L002_R SWE
GRC12117957_S GBR
GRC13193743_S12_L00 GBR
GRC15616706_S69_L00 NOR
GRC14349588_S BRA
GRC14349583_S51_L00 BRA
GRC14349601_S24_L00 BRA
HG01565 PER*
GRC14443124_S7_L00 PER
HG02271 PER*
GRC14349598_S5 COL
GRC14349600_S PER
GRC14349595_S BRA
SQVCW ARG
TYEQC ARG
GS000019960-ASM ARG*
GS000016946-ASM ARG*
GS000016945-ASM ARG*
N87FK8 ARG
GRC14349596_S BRA
HG01926 PER*
HG02265 PER*
HG01967 PER*
NA19729 MEX*
NA19774 MEX*
LP6005443-DNA_A12 MEX
LP6005677-DNA_D01 MEX
HG01139 COL*
HG01142 COL*
SS6004476 BRA
NA19783 MEX*
NA19735 MEX*
NA19732 MEX*
LP6005441-DNA_E10 MEX
NA19786 MEX*
LP6005519-DNA_A03 MEX
NA19682 MEX*
NA19664 MEX*
LP6005519-DNA_D01 ARG
LP6005443-DNA_G11 MEX
HG01124 COL*
HG01950 PER*
HG01938 PER*
HG01977 PER*
GS000019961-ASM ARG*
GRC14349593_S BRA
HG01961 PER*
GRC14349599 BRA
GRC14349587_S16_L00 ECU
HG01920 PER*
N8A2QN ARG
M39DJ ARG
UCNEN ARG
HG02090 PER*
LP6005677-DNA_E01 PER
GS000016940-ASM ARG*
HG01979 PER*
HG02277 PER*
HG02285 PER*
HG02299 PER*
T4WQV ARG
GS000019989-ASM ARG*
6QHWE ARG
LP6005441-DNA_G06 BRA
GRC14349592_S BRA
GRC14349590_S PER
GS000017077-ASM ARG*
HG01974 PER*
HG02104 PER*
HG02259 PER*
GRC14349594_S13_L00 BOL
HG01892 PER*
HG02304 PER*
HG02146 PER*
GS000016942-ASM ARG*
GS000020273-ASM ARG*
GS000016951-ASM ARG*
HG02291 PER*
HG01923 PER*
LD4PC ARG
GS000020274-ASM ARG*
EKEFB ARG
GRC14443115_S20_L00 PER
Scale (SNP number)
1
5
10
20
50
